# Crack propagation analysis using a hybrid 2D finite element–peridynamics framework with a residual-based quasi-static solver

**DOI:** 10.1007/s10704-026-00933-y

**Published:** 2026-07-10

**Authors:** Laxman Khanal, Mijia Yang

**Affiliations:** Department of Civil, Construction and Environmental Engineering, North Dakota State University, Fargo, ND 58104 USA

**Keywords:** Peridynamics, FE–PD coupling, Crack branching, Residual-based static solver, Dynamic relaxation, Crack propagation, Stress concentration

## Abstract

This paper presents a hybrid finite element–peridynamics (FE–PD) framework for two-dimensional fracture simulation. The framework embeds a peridynamic fracture zone within surrounding finite element domains through a modified volume-based (VL) coupling scheme, enabling accurate representation of crack initiation and propagation while retaining the computational efficiency of finite element analysis in the elastic far field. A major contribution of this work is the development of a residual-based dynamic relaxation solver (RBDR) for quasi-static analyses of hybrid FE–PD coupled systems. Unlike adaptive dynamic relaxation (ADR) methods, which rely on artificial damping and fictitious mass to suppress oscillations, the proposed solver is entirely damping-free and achieves equilibrium by directly minimizing the residual force field through a Richardson-type pseudo-time iteration. The solver is matrix-free, reaches higher accuracy, and 23 times faster in wall-clock time than the standard Adaptive Dynamic Relaxation method. The framework is validated against the classical Kirsch stress concentration problem, where the ABAQUS FE model recovers the analytical SCF of 3.0 and the hybrid FE–PD model reproduces the correct quantitative distribution, except at the hole surface. The hybrid method with both RBDR and ADR is then applied to crack propagation problems under Mode I tensile loading, reproducing the crack morphology and global kinetic energy evolution of a reference PD-only model with high accuracy. For the crack propagation case under quasi-static loading at four times the dynamic load level solved through the hybrid method with RBDR, crack branching does not occur, consistent with classical fracture mechanics theory that inertia is the driven factor for dynamic instability.

## Literature review

Fracture has long been recognized as a critical concern in engineering applications, motivating extensive efforts to understand material failure mechanisms and mitigate potential risks. Early contributions by Inglis and Griffith laid the foundation of fracture analysis, while Irwin extended Griffith’s theory through the concept of energy release rate (Anderson 2005). Compared with traditional strength-based design approaches, fracture mechanics provides a different framework for structural assessment. However, classical fracture mechanics suffers from inherent limitations, such as the requirement to predefine crack locations and the assumption that the fracture process zone remains small relative to the characteristic dimensions of the structure (Irwin and De Wit 1983; Irwin 1968).

The description of large and complex systems containing internal damage is of significant scientific interest. Damage evolution in real materials exhibits a non-local character due to the presence of intrinsic material length scales associated with internal microstructures. As a result, purely local continuum theories are often inadequate for capturing such behavior, which motivates the development and application of non-local theories (Pernatii et al. 2023).

To address the limitations of classical fracture mechanics and enable the modeling of crack initiation and growth, numerous numerical approaches have been proposed. Cohesive crack models establish relationships between crack opening displacement and cohesive tractions resisting separation (Barenblatt 1962; Ruiz et al. 2001), and are commonly implemented within finite element (FE) frameworks using interface elements or contact formulations. The partition of unity finite element method (PUFEM), introduced by (Melenk and Babuška 1996), provides a general enrichment strategy that was later extended to fracture simulations by Belytschko and co-workers (Belytschko and Black 1999; Moes et al. 1999), leading to the development of the extended finite element method (XFEM). XFEM allows discontinuities to be represented independently of the mesh topology and avoids remeshing procedures (Cox 2009; Meschke and Dumstorff 2007).

Peridynamics theory (Silling 2000), formulated as an alternative framework to classical continuum mechanics, enables the modeling of fracture processes such as crack propagation and bifurcation while providing realistic representations of crack paths and propagation velocities (Bobaru and Zhang 2015). As a fundamentally non-local theory, peridynamics introduces a finite interaction distance, referred as the horizon δ (Silling 2000; Silling and Askari 2005), which governs the range of long-range forces and defines the degree of non-locality.

In the bond-based peridynamics formulation (Silling et al. 2007), interaction forces depend on the collective deformation of all bonds within a spherical neighborhood of radius δ. When the deformation exceeds a prescribed threshold, bonds are irreversibly broken, representing material damage and crack formation (Silling 2003). Although this formulation allows for natural treatment of discontinuities, accurate representation of continuous deformation fields requires high spatial resolution, making peridynamics simulations computationally expensive compared to local methods.

The treatment of boundary conditions in peridynamics also differs fundamentally from classical continuum theories. Since the variational formulation does not lead to natural boundary conditions (Weckner and Abeyaratne 2005), external forces must be applied over finite regions rather than along surfaces or lines (Silling and Lehoucq 2008). This can introduce softening effects near boundaries when material points lie within one horizon distance from the domain edges (Zaccariotto et al. 2015).

Despite its advantages for fracture modeling, the high computational cost of peridynamics has motivated the development of coupling strategies that combine local and non-local formulations (Hong and Bathe 2005; Liu and Gu 2000). Several benchmark fracture problems, such as the mixed-mode double-edge-notched concrete specimen tested by (Nooru-Mohamed et al. 1993), have been extensively studied using a wide range of numerical approaches, including smeared-crack models, gradient-enhanced plasticity, cohesive crack formulations, XFEM, and hybrid analytical–numerical techniques (De Borst 1997; Di Prisco et al. 2000; Gasser and Holzapfel 2006; Patzák and Jirásek 2004; Pivonka et al. 2004; Réthoré et al. 2010; Unger et al. 2007). In recent years, coupling local and non-local theories have been widely studied. In such hybrid frameworks, non-local regions are employed in areas where discontinuities exist or are expected to form, while local models are used elsewhere to reduce computational cost and facilitate the application of conventional boundary conditions.

Since approximately 2015, the field of FE–PD coupling has seen considerable advances beyond the foundational works of the early 2010s. The research group of Galvanetto, Zaccariotto, Shojaei, and co-workers at the University of Padova has made particularly significant contributions. (Galvanetto et al. 2016) proposed an effective approach to couple FE meshes with PD grids for static equilibrium problems. (Zaccariotto et al. 2017) introduced an enhanced coupling that improved interface accuracy, and (Shojaei et al. 2017) developed a switching-based coupling using classical elasticity to eliminate ghost forces. (Zaccariotto et al. 2018) presented a comprehensive FE–PD coupling framework applicable to dynamic fracture. (Ongaro et al. 2021) addressed overall equilibrium consistency in PD–continuum coupling, and (Ni et al. 2024) implemented GPU-accelerated hybrid FE–PD models for hydro-mechanical cracking problems. A complementary line of research has developed “fast solvers” for PD models as an alternative route to efficiency. (Jafarzadeh et al. 2022) introduced a fast convolution-based method (FCBM) using FFT that reduces PD complexity from O(N^2^) to O(N·log_2_N), with dramatic speedups for elasticity and dynamic brittle fracture including crack branching. The associated open-source code PeriFast/Dynamics (Jafarzadeh et al. 2024) extends FCBM to explicit dynamic simulation. For problems where damage is distributed across the full domain, such fast PD solvers may be more efficient than FE–PD coupling; for localized fracture in large elastic domains, coupled approaches avoid applying the nonlocal PD operator over the elastic exterior, providing a natural efficiency advantage. The present paper builds on the VL-coupling approach of (Liu and Hong 2012b) and introduces a modified formulation that improves energy consistency at the interface.

Various FE–peridynamics coupling strategies have been proposed, including embedded peridynamics elements (Lall et al. 2010; Macek and Silling 2007), overlapping domain formulations (Kilic and Madenci 2010b), sub-model techniques (Agwai et al. 2009), and energy-based morphing approaches (Lubineau et al. 2012). To address the computational intensity of peridynamics, (Liu and Hong 2012a) proposed a hybrid modeling approach that directly couples discretized peridynamics with the finite element method (FEM). The domain is partitioned such that subregions where failure is expected are modeled using peridynamics, while the remainder of the domain is modeled with FEM to maintain computational efficiency. These subregions are bridged by interface elements containing embedded peridynamics nodes that facilitate the calculation of coupling forces defined as the interaction between embedded nodes and peridynamics nodes outside the interface element. Two distinct coupling schemes, VL-coupling (Volume-Coupling) and CT-coupling (Contact-Coupling), exist in literature, which differ in their method of force distribution (Liu and Hong 2012b). In VL-Coupling, forces are distributed throughout the element’s volume, the FE nodes at the actual subregion interface only receive partial coupling forces, leading to slightly overestimated displacements at these interfaces (Liu and Hong 2012b). In contrast, the CT-coupling scheme treats the boundary between subregions as a contact surface and distributes coupling forces only to the FE nodes located on the interface segment, producing results that were almost identical to classical quasi-static solutions (Liu and Hong 2012b).

In this paper, the introduction and literature review are summarized in Sect. 1, and Sect. 2 describes the fundamental concepts of peridynamics formulations, stress definition, and bond breakage in peridynamics. A hybrid two-dimensional FE–PD framework is then developed in Sect. 3 to couple the finite element (FE) and peridynamics (PD) domains through a modified VL-coupling scheme. In the proposed formulation, coupling forces arising from the interactions of embedded peridynamics nodes are transferred to the finite element interface using a volume-based distribution governed by the shape functions of the interface element. Unlike the conventional VL-coupling approach, the proposed modification enhances the consistency of force transfer across the FE–PD interface while retaining the simplicity and efficiency of the volume-based formulation. This improved coupling strategy ensures stable displacement compatibility between the FE and PD subregions, thereby enabling accurate prediction of crack initiation and propagation within the peridynamics region while maintaining the computational efficiency of the surrounding FE domains. Furthermore, in Sect. 4, a novel Residual-Based Dynamic Relaxation solver (RBDR) is introduced for quasi-static peridynamics problems, which operate without artificial damping. In Sect. 5, the two main contributions of this paper are therefore: (i) the RBDR quasi-static solver, which offers substantial convergence advantages over ADR and is validated on the Kirsch problem; and (ii) the modified VL-coupling FE–PD formulation. This method, along with the adaptive dynamic relaxation (ADR) method by (Kilic and Madenci 2010a), is evaluated within the PD domain to conduct a comparative study on convergence behavior, kinetic energy dissipation rates, and the decay of residual forces. Prior to the quasi-static crack branching simulation, the modified VL-coupling FE–PD framework is first validated against the dynamic crack branching benchmark using both the FE–PD hybrid model and a PD-only domain, confirming the accuracy and stability of the coupling scheme under dynamic conditions. Building on this validated framework, the quasi-static crack branching problem is then investigated using the modified VL-coupling FE–PD formulation in conjunction with the RBDR solver. Section 6 presents the concluding remarks, summarizes the principal findings, and outlines directions for future research.

## Bond-based peridynamics and its stress and energy density definitions

### Peridynamics theory

Peridynamics describes the motion of material points through integral equations. For a material point *x* in the reference configuration at time *t*, the equation of motion is given by (Liu and Hong 2012a; Silling 2000):

1 $$ \rho \ddot{u}\left( {x,t} \right) = \mathop \int \limits_{{H_{x} }} f\left( {\eta ,\xi } \right)dV_{x^{\prime}} + b\left( {x,t} \right) $$

Here, ρ represents the mass density of the material, which characterizes how much mass is contained within a unit volume and directly influences the inertial response of the body. The term $\ddot{u}\left(x,t\right)$ denotes the acceleration vector at a material point *x* at time *t*, describing the dynamic motion of that point under applied forces. The region ${H}_{x}$ defines the neighborhood, or horizon, of point *x*; it contains all material points *x′* that interact nonlocally with *x* within a finite distance. The function $f\left(\eta ,\xi \right)$ is the pairwise force density function that governs the interaction between two material points, where *η* typically represents the relative displacement vector and *ξ* the relative position vector in the reference configuration. The quantity, $d{V}_{x}$, is the differential volume element associated with the interacting point *x′*, ensuring that the force contributions are integrated over the entire neighborhood. Finally, b(x,t) denotes the body force density field, which accounts for externally applied forces per unit volume, such as gravity or other distributed loads acting on the material point.

### Bond-based peridynamics formulation

In bond-based peridynamics, the pairwise force vector is expressed as (Liu and Hong 2012a):

2 $$ f\left( {\eta ,\xi } \right) = f\left( {\eta ,\xi } \right)\frac{{\left( {\eta + \xi } \right)}}{{\left| {\left| {\eta + \xi } \right|} \right| }} $$

For a microelastic brittle material model, the bond force is given by:

3 $$ f\left( {\eta ,\xi } \right) = cs\left( {\eta ,\xi } \right)\mu \left( {\eta ,\xi } \right) $$

where *c* is the micromodules constant, $s\left(\eta ,\xi \right)$ is the stretch of the bond, and *μ* is the bond failure function.

For 2D plane stress condition, the micromodules constant is (Seleson et al. 2024).

4 $$ c = \frac{9E}{{\pi h\delta^{3} }} $$

where, *E* elastic modulus of material, *h* is the thickness, and δ represents the horizon size.

The critical bond stretch *s*_*0*_ for bond failure is related to the critical fracture energy release rate ${G}_{0}$ by (Seleson et al. 2024):

5 $$ s_{0} = \sqrt {\frac{{4\pi G_{0} }}{9E\delta }} $$

The bond failure function $\mu \left(t,\eta ,\xi \right)$ at any time instant* t* is defined as:

6 $$ \mu \left( {t,~\eta ,\xi } \right) = \left\{ {\begin{array}{*{20}c}    {1~~~if~s\left( {\eta ,\xi } \right) < s_{0} ~}  \\    {0~~~~~otherwise}  \\   \end{array} } \right. . $$

### Damage model and failure criterion

The damage at a material point is quantified by the damage index *φ(x, t)*, defined as the ratio of broken bonds (Liu and Hong 2012a; Seleson et al. 2024):

7 $$ \varphi \left( {x_{i} ,t} \right) = 1 - \frac{{\left[ {\Sigma \mu \left( {\eta ,\xi } \right)V_{J} } \right]}}{{\left[ {\Sigma V_{J} } \right]}} $$

where the summations are over all neighbors *J* within the horizon of point *i*. Complete failure occurs when *φ* approaches 1.0 (Figs. 1, 2, 3, 4, 5, 6, 7, 8, 9 and 10).

**Fig. 1:**
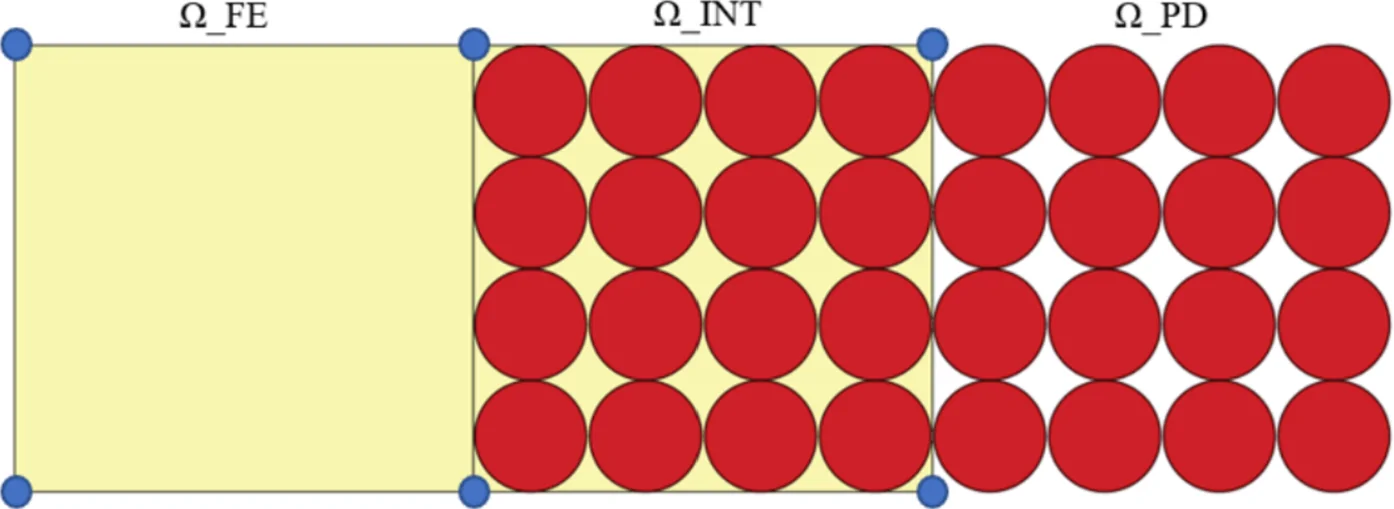
Conceptual diagram of the 2D FE–PD coupled domain

**Fig. 2:**
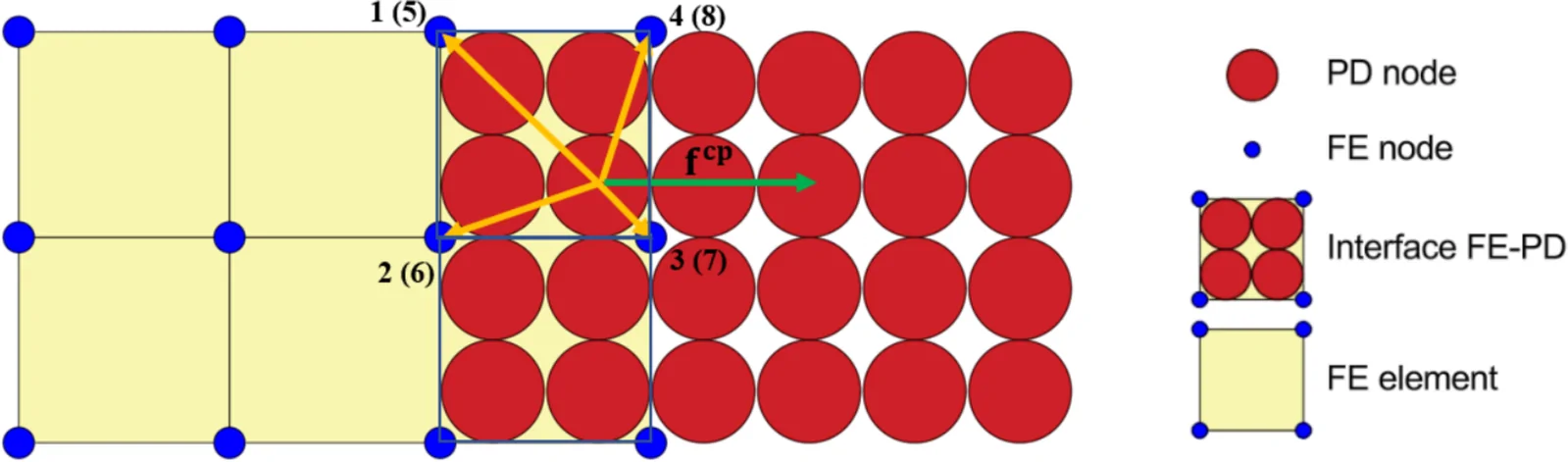
The VL coupling scheme distributes the coupling force ${\mathrm{f}}^{\mathrm{c}\mathrm{p}}$ among the FE nodes of the interface element

**Fig. 3:**
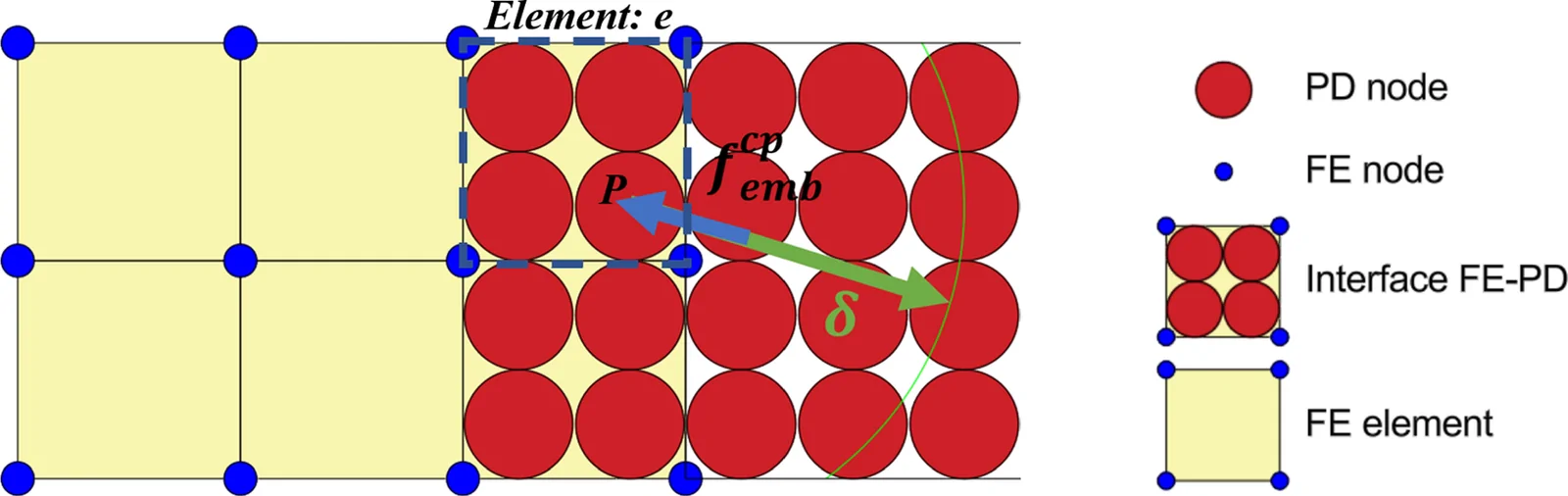
Computation of the coupling force ${\mathbf{f}}^{\mathbf{c}\mathbf{p}}$ on an embedded node from its interactions with PD nodes located outside the interface element

**Fig. 4:**
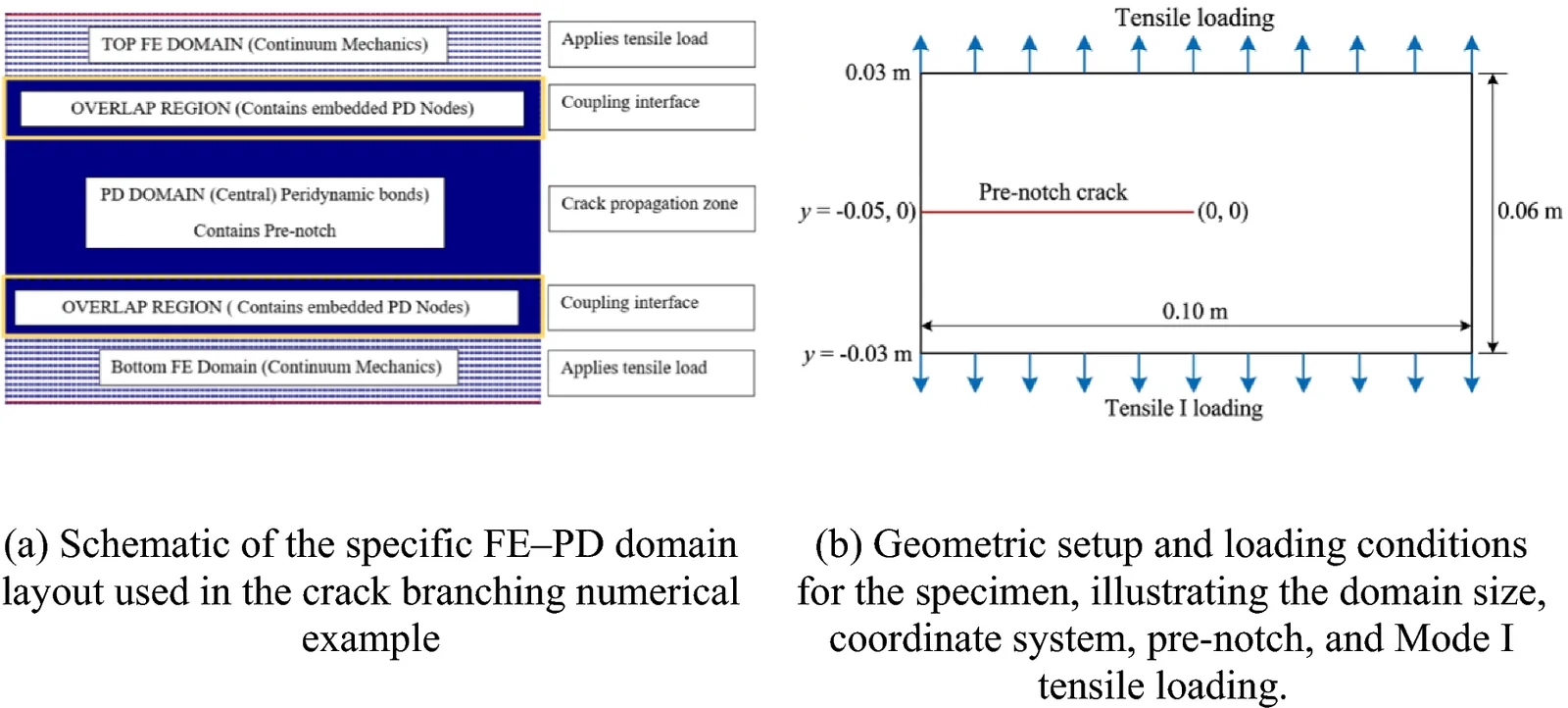
FE-PD coupling domain for crack branching.

**Fig. 5:**
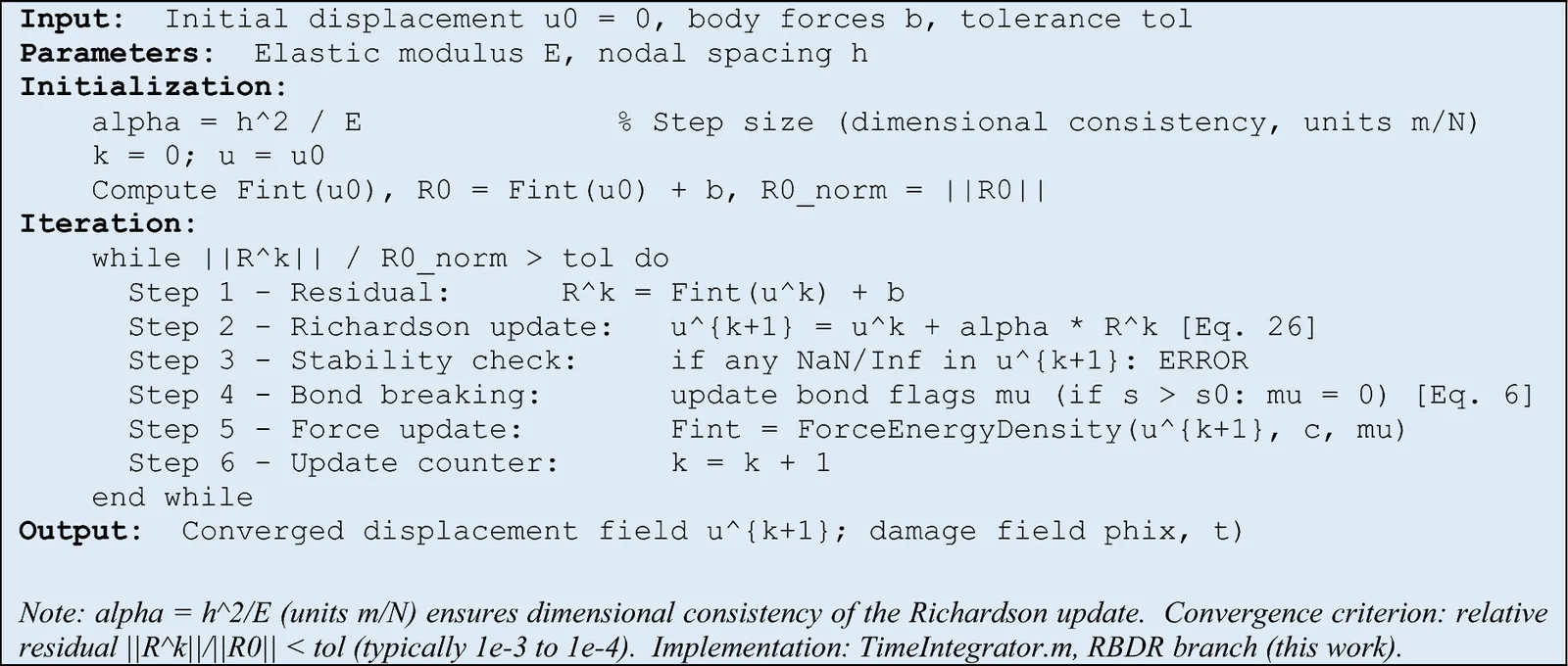
RBDR pseudocode

**Fig. 6:**
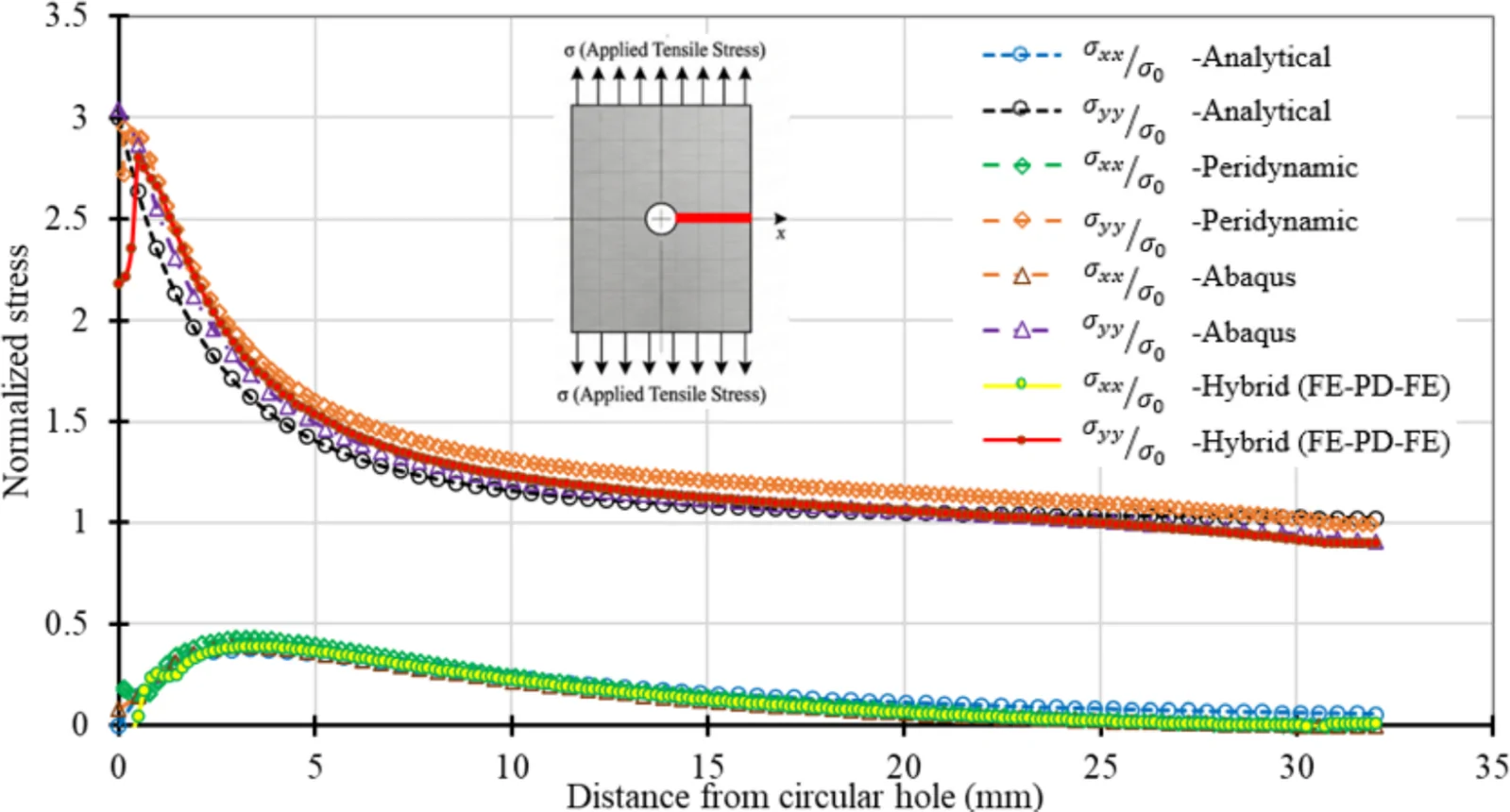
Comparison of stress concentration factor (SCF) obtained using PD, FE–PD coupling, ABAQUS FE simulation, and analytical formulation

**Fig. 7:**
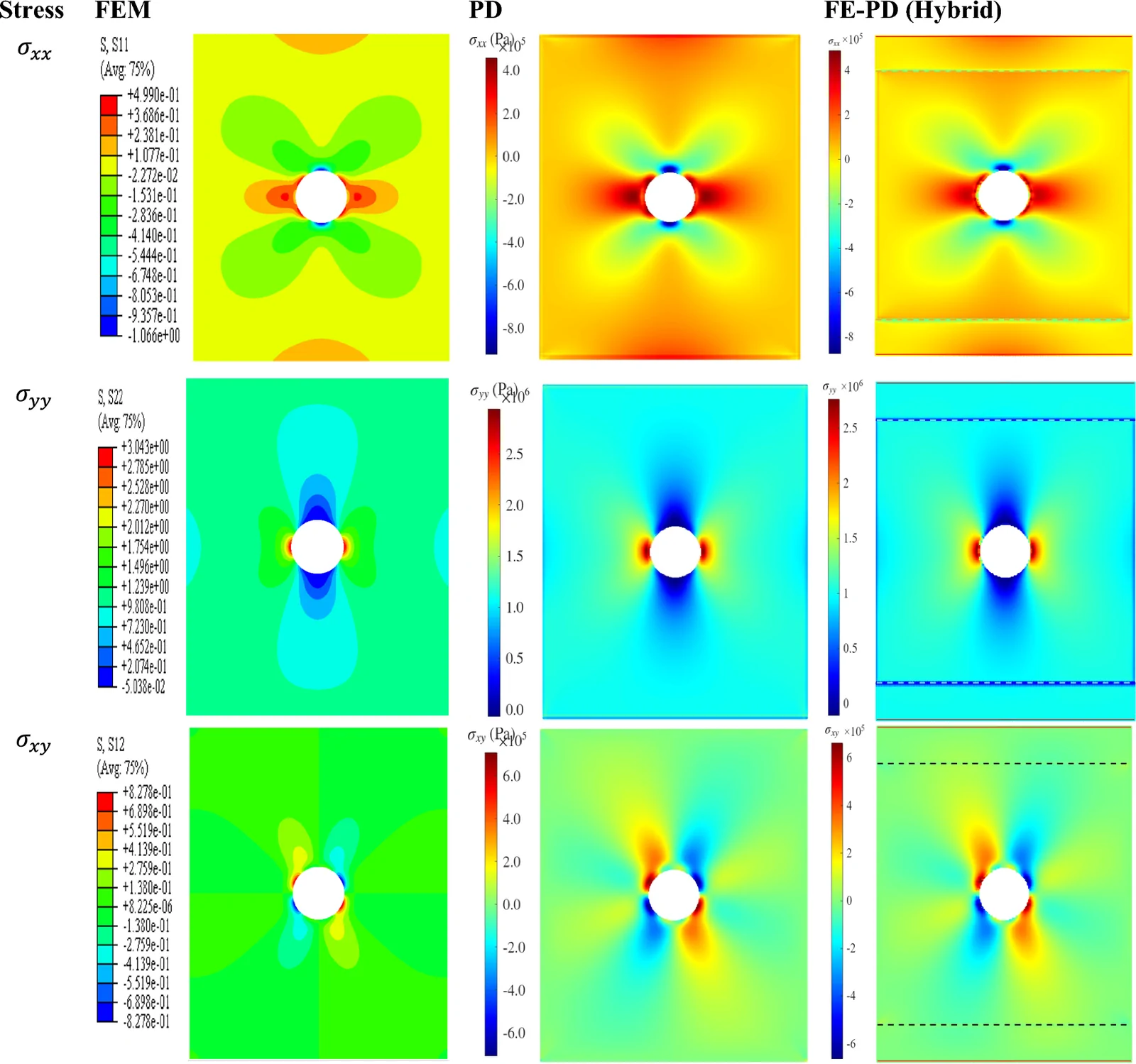
Comparison of stress distributions (${\sigma}_{xx},{\sigma}_{yy}, and{ \sigma }_{xy}$) obtained using the finite element method (FE), peridynamic model (PD), and the hybrid FE–PD formulation

**Fig. 8:**
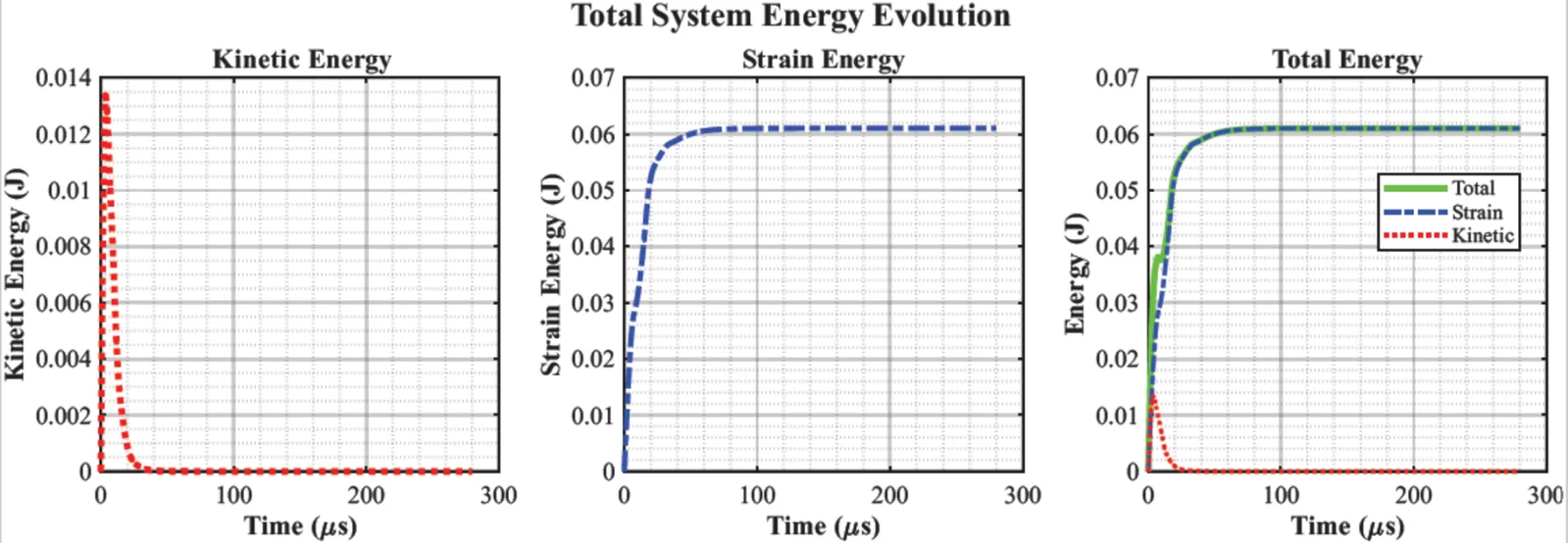
Convergence behavior of the hybrid FE–PD model using the RBDR method, showing the decay of kinetic energy and stabilization of strain and total energies

**Fig. 9:**
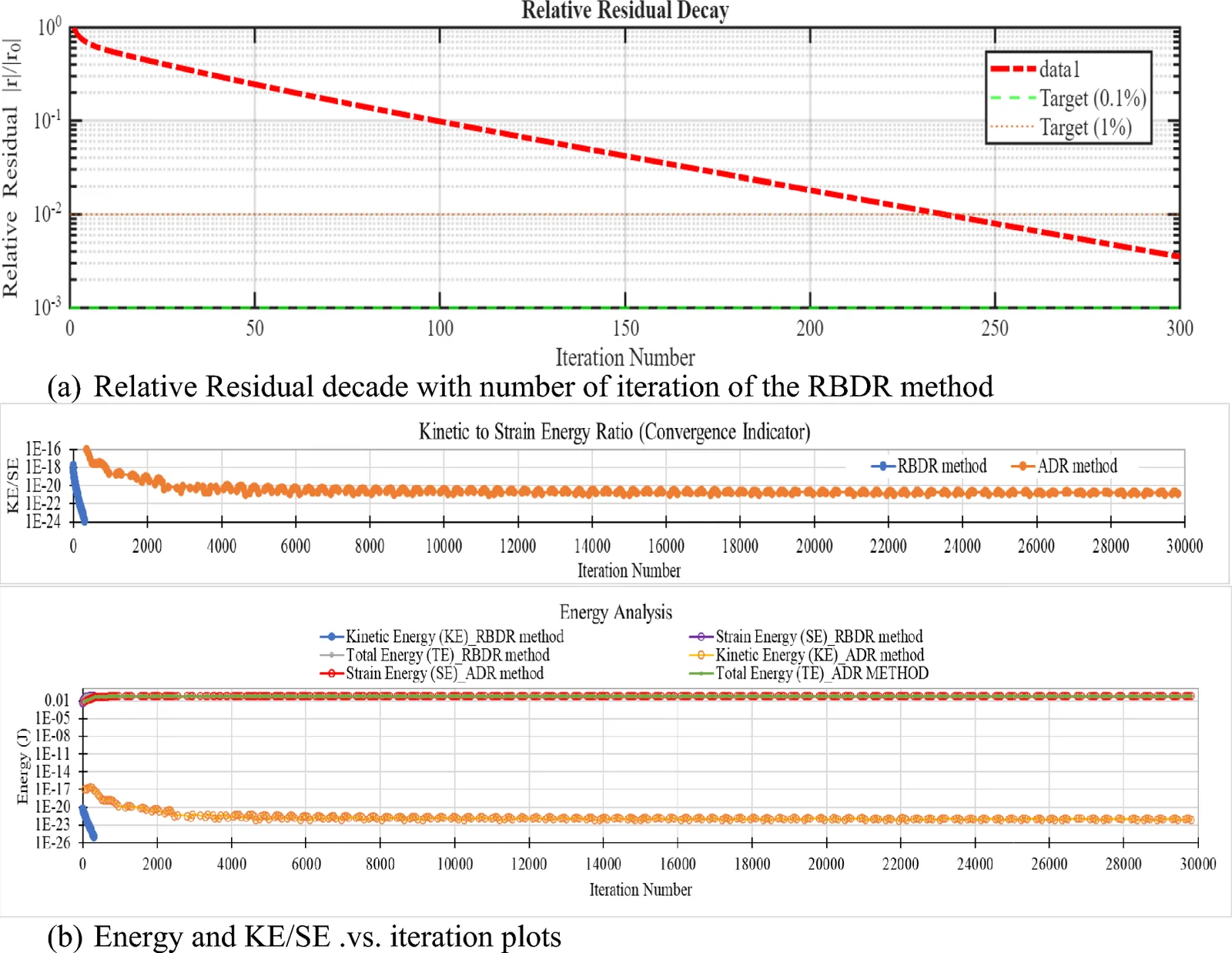
Dynamic relaxation by the RBDR VS ADR method. a Relative Residual decade with number of iteration of the RBDR method. b Energy and KE/SE.vs. iteration plots

**Fig. 10:**
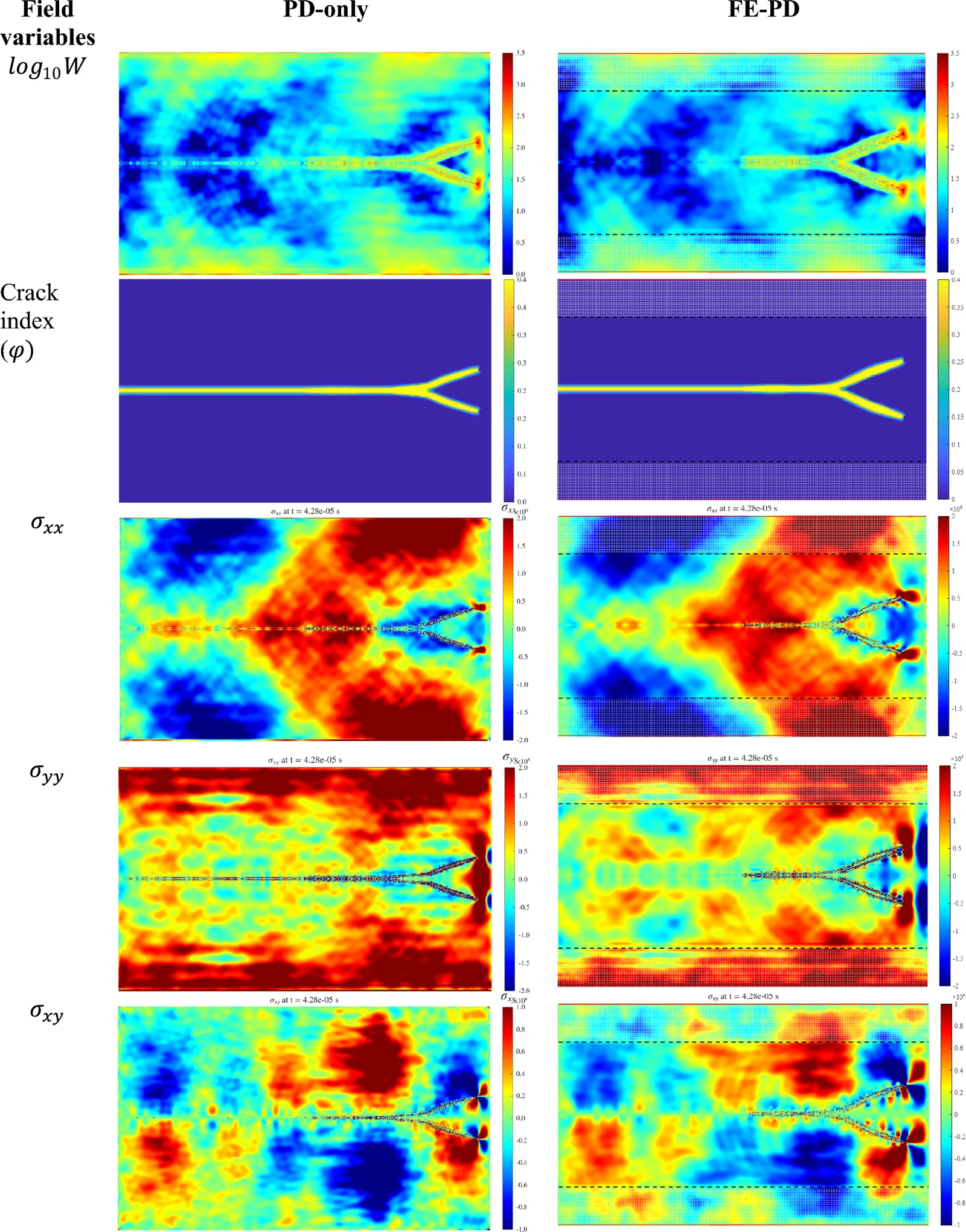
Mechanical response quantities obtained from the PD-only and hybrid FE–PD models, including both inertial and strain energy effects during crack branching

### Peridynamics virial stress

**Fig. 11:**
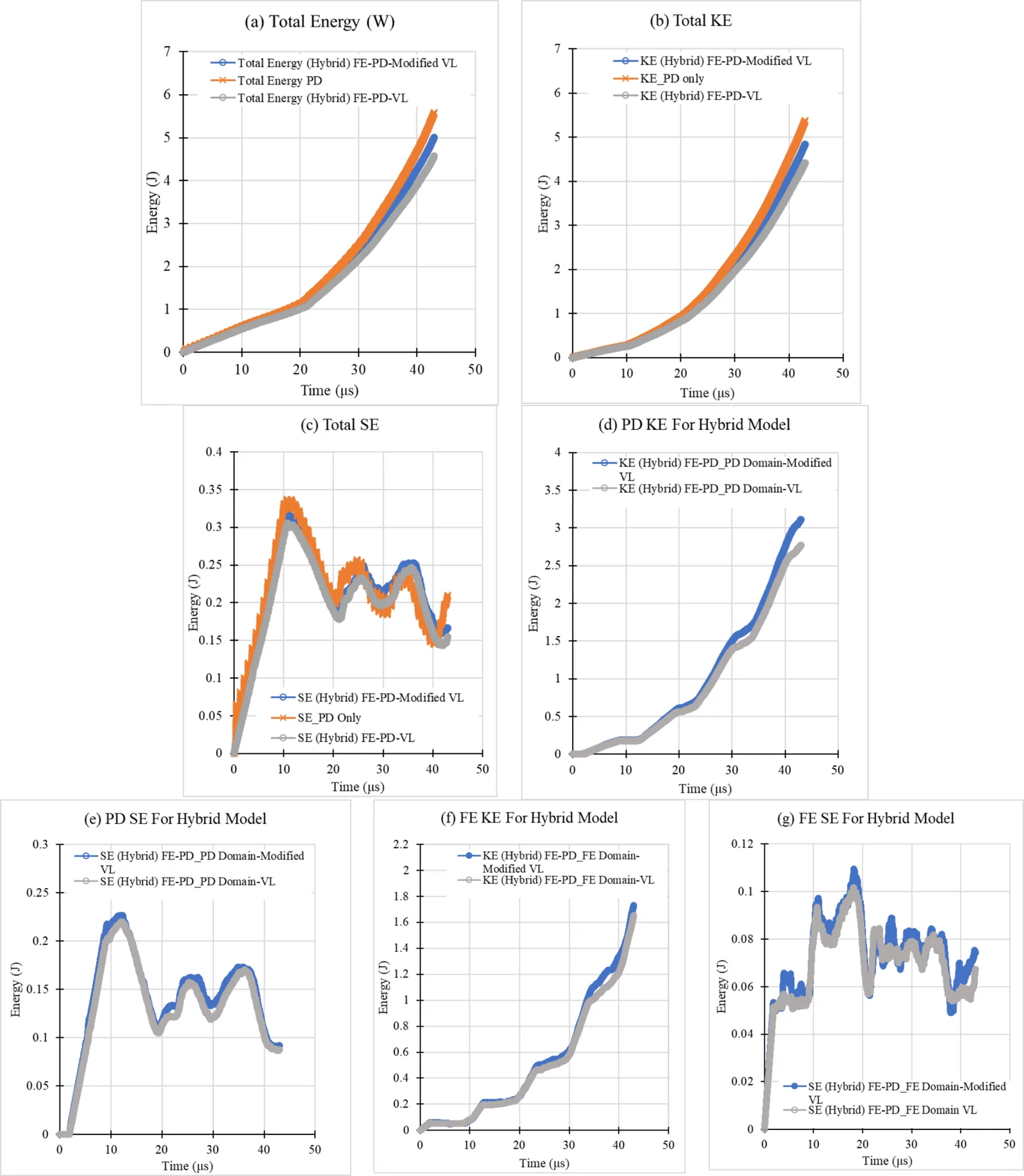
Comparison of energy responses between the pure Peridynamics (PD) and hybrid FE–PD models based on the modified VL and VL-coupling formulation, showing: **a** total system energy evolution for the hybrid FE–PD and pure PD models; **b** total kinetic energy (KE) comparison between the hybrid FE–PD and pure PD models; **c** total strain energy (SE) evolution for the hybrid FE–PD and pure PD models; **d** kinetic energy of the PD subdomain within the hybrid models; **e** strain energy of the PD subdomain within the hybrid models; **f** kinetic energy of the FE subdomains within the hybrid models; and **g** strain energy of the FE subdomains within the hybrid models

The virial stress formulation presented in this subsection is employed to compute all stress field plots in this work. The *σ*_*xx*_, *σ*_*yy*_, and *σ*_*xy*_ contour plots in Figs. 6, 9 and 11 are all computed using the push-forward formulation in Eq. (10). Mathematical Framework for the First Piola–Kirchhoff Virial Stress is defined by (Li et al. 2022). The approach provides a simpler and more computationally efficient method than the original Lehoucq-Silling formulation (Lehoucq and Silling 2008). The peridynamic stress at material point *x* is computed as

8 $$ P\left( x \right) = \frac{1}{{2\Omega_{x} }}\mathop \sum \limits_{I = 1}^{N} \mathop \sum \limits_{J = 1,I \ne J}^{N} t_{IJ} \otimes \xi_{IJ} $$

where $\xi  = x^{\prime} - x\, \text {and}\,\xi _{{IJ}}  = x_{J}  - x_{I}$ for reference bond vector *IJ*.

9 $$ t_{{IJ}}  = c\left( {\xi _{{IJ}} } \right)\frac{{\left\| {\left. {\eta _{{IJ}}  + \xi _{{IJ}} } \right\|} \right. - \left\| {\left. {\xi _{{IJ}} } \right\|} \right.}}{{\left\| {\left. {\xi _{{IJ}} } \right\|} \right.}}\frac{{\eta _{{IJ}}  + \xi _{{IJ}} }}{{\left\| {\left. {\eta _{{IJ}}  + \xi _{{IJ}} } \right\|} \right.}}V_{I} V_{J}       $$

In this formulation, ${\eta}_{IJ}={u}_{J}-{u}_{I}$ represents the relative displacement between particles *I* and *J*, defined as the difference in their displacement vectors. The term ${\Omega}_{x}$ = π δ^2^ denotes the horizon area in a two-dimensional setting, where *δ* is the horizon radius that defines the extent of nonlocal interaction around a material point. The vector ${t}_{IJ}$ corresponds to the bond force exerted by particle *J* on particle *I*, describing the pairwise interaction force along the bond connecting the two particles. The symbol ⊗ indicates the tensor (outer) product, which forms a second-order tensor from two vectors and is commonly used in stress or force state formulations (Lehoucq and Silling 2008). The parameter *c* is the micromodules constant that controls the stiffness of the bond and governs the material’s elastic response within the peridynamics framework.

The Cauchy stress at each material point is obtained by first assembling the peridynamic Virial first Piola–Kirchhoff stress *P*(*x*) (Li et al. 2022), and the nonlocal deformation gradient *F* via the weighted least-squares form. The Cauchy stress is then recovered through the standard continuum mechanics push-forward:

10 $$ \sigma = J^{ - 1} P\left( x \right)F^{T} $$

where $$J=\mathrm{d}\mathrm{e}\mathrm{t}(F)$$.

### Macroelastic energy density

For a bond-based peridynamics model under the plane stress condition, the macroelastic energy density *W* for an isotropic extension with constant stretch $\overline{s }$ is given by:

11 $$ W = \frac{{3E\overline{s}^{2} }}{2} $$

where *E* is the effective peridynamics modulus. This expression follows directly from the peridynamics constitutive relation integrated over the horizon ${H}_{x}$, and differs from the plane strain case *(*$W=\frac{8E{\overline{s} }^{2}}{5}$*)* due to the zero out-of-plane stress constraint (Seleson et al. 2024).

## Hybrid FE-PD coupling methodology

### Domain decomposition strategy

The computational domain *Ω* is partitioned into non-overlapping subregions (Liu and Hong 2012b):

12 $$ \Omega = \Omega_{FE} \cup \Omega_{INT} \cup \Omega_{PD} $$

In this domain decomposition, ${\Omega}_{FE}$ denotes the finite element subregion in which the classical finite element method is employed to model the structural response using local continuum mechanics. The region ${\Omega}_{PD}$ represents the peridynamics subregion, where the nonlocal peridynamics theory is applied, particularly to capture crack initiation and propagation without requiring additional criteria or remeshing. The interface region, ${\Omega}_{INT}$, consists of special interface elements that provide the coupling between the FE and PD subregions, ensuring compatibility of kinematics and equilibrium of forces across the transition zone so that the overall formulation remains stable and consistent. In the present implementation, the FE–PD interface is straight (rectilinear and mesh-aligned). For problems requiring curved or irregular interfaces, the shape function interpolation of Eq. (13) is geometry-independent in principle; however, accuracy for such configurations has not been verified and is left for future work.

In the numerical examples of this paper, the PD region is flanked by two FE subdomains (a sandwich arrangement), as shown in Fig. 1. The essential element of the model is a single FE–PD coupling interface that happens to be instantiated twice in this geometry. Any number of alternating FE and PD subregions could employ the same coupling interface formulation without requiring a new methodological name. The PD region contains the fracture zone, while FE regions model elastic surroundings.

### VL-coupling scheme

In the VL-coupling scheme, coupling forces on embedded PD nodes are distributed to All FE nodes of the interface element based on shape function values (Liu and Hong 2012b). This scheme treats the entire volume of the interface element as active in the coupling.

For an embedded PD node subjected to coupling force ${\mathrm{f}}^{\mathrm{c}\mathrm{p}}$, the force distributed to FE node *i* shown in Fig. 2 is

13 $$  f_{i}^{{cp}}  = N_{i} \left( {\xi ,\eta } \right)f^{{cp}} \,\left( {i = 1, \ldots ..,8} \right)  $$

The coupling force ${{\boldsymbol{f}}}^{cp}$ on an embedded node is calculated from its interactions with PD nodes outside the interface element shown in Fig. 3.

14 $$ f_{emb}^{cp} = \mathop \sum \limits_{{\left( {J[ PD} \right)}} f\left( {\xi_{J} ,\eta_{J} } \right)V_{J} $$

where the summation is over all PD nodes *J* outside the interface element but within the horizon of the embedded node.

For displacement compatibility, an embedded PD node *p* located in FE element *e* has

15 $$ u_{p}^{PD} = \mathop \sum \limits_{i = 1}^{4} N_{i} \left( {\xi_{p} ,\eta_{p} } \right)u_{{i_{e} }}^{FE} $$

where ${N}_{i}$ is a 4-node quadrilateral element shape function, given natural coordinates (*ξ*, *η*) ∈ [− 1, 1].

### Modified VL-coupling scheme

In an interface element, there is at least one or multiple peridynamics (PD) particles embedded. The motion of each embedded PD particle is assumed to follow the finite element interpolation, ensuring kinematic compatibility between the local (FE) and nonlocal (PD) domains. Based on this assumption, the governing relationships are derived systematically, including the displacement compatibility relation, the mass consistency relation, and the force transfer relation, thereby ensuring energy consistency and mechanical equilibrium between the coupled domains.

Considering a two-dimensional four-node quadrilateral (Q_4_) finite element with four nodes and two degrees of freedom per node, the element displacement vector is

16 $$ U_{e} = \left[ {\begin{array}{*{20}c} {u_{1} } & {v_{1} } & {u_{2} } & {v_{2} } & {u_{3} } & {v_{3} } & {u_{4} } & {v_{4} } \\ \end{array} } \right]^{T} $$

The standard bilinear shape functions in natural coordinates $\left(\xi ,\eta \right)$ are given by ${N}_{1}\left(\xi ,\eta \right),{N}_{2}\left(\xi ,\eta \right),{N}_{3}\left(\xi ,\eta \right)$, and ${N}_{4}\left(\xi ,\eta \right)$. For a peridynamic (PD) particle embedded at $\left({\xi}_{p},{\eta}_{p}\right)$, the shape functions are evaluated as ${{N}_{i}}^{p}={N}_{i}\left({\xi}_{p},{\eta}_{p}\right)$. Kinematic compatibility is enforced by assuming that the PD particle follows the FE interpolation field, leading to the displacement relation.

17 $$ u_{p} = \mathop \sum \limits_{i = 1}^{4} N_{i}^{p} u_{i} , v_{p} = \mathop \sum \limits_{i = 1}^{4} N_{i}^{p} v_{i} $$

In matrix form:

18 $$ U_{p} = \left[ {\begin{array}{*{20}c} {N_{1}^{P} } & 0 & {N_{2}^{P} } & 0 & {N_{3}^{P} } & 0 & {N_{4}^{P} } & 0 \\ 0 & {N_{1}^{P} } & 0 & {N_{2}^{P} } & 0 & {N_{3}^{P} } & 0 & {N_{4}^{P} } \\ \end{array} } \right]U_{e} $$

If

19 $$ H_{p} = \left[ {\begin{array}{*{20}c} {N_{1}^{P} } & 0 & {N_{2}^{P} } & 0 & {N_{3}^{P} } & 0 & {N_{4}^{P} } & 0 \\ 0 & {N_{1}^{P} } & 0 & {N_{2}^{P} } & 0 & {N_{3}^{P} } & 0 & {N_{4}^{P} } \\ \end{array} } \right] $$

Then,

20 $$ U_{p} = H_{p} U_{e} $$

where ${H}_{p}$ is the interpolation matrix constructed from ${N}_{i}^{p}$.

The PD particle mass is defined as ${m}_{p}=\rho {V}_{p}$, and using ${U}_{p}={H}_{p}{U}_{e}$, the particle kinetic energy becomes

21 $$ T_{p} = \frac{1}{2}\dot{U}_{e}^{T} H_{p}^{T} m_{p} H_{p} \dot{U}_{e} $$

Enforcing energy equivalence between the embedded PD particle and the finite element yields the consistent mass contribution ${M}_{e}={H}_{p}^{T}{m}_{p}{H}_{p}$. For multiple embedded particles, this generalizes to ${M}_{e}=\sum_{p}{H}_{p}^{T}{m}_{p}{H}_{p}$*.* Since ${m}_{p}=\rho {V}_{p}$, mass conservation further requires the volume consistency condition ${V}_{e}=\sum_{p}{V}_{p}$, ensuring full energy and mass compatibility between the local *FE* and nonlocal *PD* descriptions.

In the *FE* subregion, a lumped mass matrix is adopted, and the internal nodal forces are assembled from element contributions as

22 $$ F_{i}^{{\mathrm{int}}} = \mathop \sum \limits_{e} K^{\left( e \right)} U^{\left( e \right)} $$

where, ${K}^{(e)}$ is the element stiffness matrix and ${U}^{(e)}$ is the element displacement vector. The equation of motion for node *I* is written as

23 $$ M_{i} \ddot{U}_{i} = F_{i}^{ext} + F_{i}^{{\mathrm{int}}} $$

where, ${M}_{i}$ is the lumped nodal mass; ${F}_{i}^{ext}$ is the external force at the node. Because the mass matrix is diagonal, the system can be advanced explicitly without global matrix factorization.

Coupling between the FE and PD subregions is achieved through an interface element. Within each interface element, several PD nodes are embedded to compute interaction forces between the embedded PD nodes and the surrounding PD nodes in the nonlocal region. These interaction forces are defined as coupling forces.

Each embedded PD node represents a material volume inside the interface element. However, embedded PD nodes do not introduce additional global degrees of freedom of the FE domain; their displacements are interpolated from the FE field using the shape functions of the interface element. In standard finite element practice, the accuracy of field quantities at any interior point within an element depends on the density of the interpolation basis—that is, the number and distribution of nodes through which the field is reconstructed. The embedded PD nodes play an analogous role: each PD node inside an interface element is assigned a displacement interpolated from the surrounding FE nodal values via shape functions, and the coupling forces computed at those PD nodes are redistributed back to the FE nodes through the same shape function weights. Consequently, increasing the number of embedded PD nodes within an interface element is functionally equivalent to locally refining the interpolation field inside that element. The more embedded nodes that are present, the more accurately the nonlocal PD force density is sampled and distributed across the interface, in the same way that mesh refinement improves the resolution of field gradients in classical FEM. When only a single PD node is embedded, the coupling force passes through a single interpolation point, which can concentrate force transfer and reduce accuracy when the horizon spans multiple neighboring PD nodes. Embedding four PD nodes per element distributes coupling forces more uniformly via shape function weighting, improving the approximation of nonlocal force transfer and reducing artificial stress oscillations in the coupling zone. This is consistent with the principle that a denser sampling of the nonlocal interaction field within the interface region yields a more accurate discrete representation of the continuous PD force density, without altering the underlying FE mesh or introducing new global unknowns. The choice of four embedded nodes per interface element therefore represents a deliberate and physically motivated discretization decision rather than an arbitrary parameter, providing sufficient resolution of the PD force field within each element for the mesh spacings employed in this study.

### Implementation of the coupling models in PDMATLAB2D

A two-dimensional hybrid Finite Element–Peridynamic (FE-PD) fracture simulation framework was implemented in PDMATLAB2D following the Modified VL-coupling methodology (Liu and Hong 2012b; Seleson et al. 2024). The peridynamics (PD) domain occupies the central fracture-containing region and is discretized on a meshfree node grid, where each node interacts nonlocally with all neighbor nodes within a prescribed horizon *δ* through the Generalised Prototype Microelastic Brittle (GPMB) constitutive model. Bond-level force densities and strain energy densities are computed via ForceEnergyDensity, micromodulus *c* and critical stretch *s*_*0*_ are derived from the macroscopic Young’s modulus *E* and fracture energy *G*_o_ via PDBondConstants, and bonds are irreversibly severed whenever their stretch exceeds *s*_*0*_. Two finite element sub-domains—one above and one below the PD region—are assembled as shown in Fig. 4 with full stiffness and consistent mass matrices by BuildFEDomainComplete, and are coupled to the PD layer through overlapping interface zones where embedded PD nodes are kinematically constrained to follow the FE displacement field interpolated by SetupFEPDCoupling. Time integration proceeds by TimeIntegrator_FE_PD, which performs a coupled Velocity Verlet–Newmark scheme. PD accelerations are updated from bond forces plus transferred interface forces, FE domains are advanced by central-difference Verlet, and FE-to-PD displacement compatibility is re-enforced at every step, with the alternative TimeIntegrator providing standard Verlet or Adaptive Dynamic Relaxation (ADR/RBDR) for PD-only runs. Pre-existing cracks are introduced geometrically via PreNotch using segment-intersection tests, a no-fail mask protects overlap regions from spurious bond breakage, and post-processing exports full energy histories (kinetic, strain, system total) to Excel, including displacements, damage index φ, and stress components. 

## Residual-based dynamic relaxation (RBDR) method and its comparison with ADR

Peridynamics simulations of quasi-static problems are commonly performed using dynamic relaxation techniques, in which artificial inertia and damping are introduced to drive the system toward equilibrium ((Kilic and Madenci 2010a). In the present work, the explicit Velocity Verlet–Newmark time integration scheme is used on the dynamic crack branching analysis (Sect. 5.3.1). In contrast, a quasi-static crack branching analysis is performed using the RBDR method (Sect. 5.3.3). Although effective for quasi-static PD, ADR-type approaches require careful tuning of mass and damping parameters and may obscure the underlying static nature of the problem.

In this work, a residual-based explicit equilibrium solver is presented for peridynamics models that avoid artificial mass, damping, and stiffness matrix construction. The method employs a Richardson-type pseudo-time iteration in which nodal displacements are updated directly in the direction of the force residual.

### Residual-based explicit equilibrium solver

For static equilibrium problems, inertial effects are absent, and Eq. (1) reduces to

24 $$ F_{{\mathrm{int}}} \left( u \right) + b = 0 $$

where ${F}_{int}$ represents the discretized internal force vector assembled from peridynamic interactions. Let us reformulate the static equilibrium equations as

25 $$ R\left( u \right) = F_{{\mathrm{int}}} \left( u \right) + b $$

The objective is to find *u* such that $R\left(u\right)=0$.

A Richardson-Type Update is proposed to solve the above residue:

26 $$ u^{k + 1} = u^{k} + \alpha R\left( {u^{k} } \right) $$

where α is a positive scalar controlling the update magnitude. This is equivalent to a classical Richardson iteration applied to the nonlinear equilibrium equations. Unlike implicit methods, no Jacobian or stiffness matrix is constructed. The parameter α is chosen based on dimensional consistency. In bond-based PD, the residual force has units of N. To produce a displacement update (units m), α must carry units m^4^/N per unit volume. The bond stiffness is proportional to *EA*/*h*, so the force volume density at each PD node is proportional to $${\raise0.7ex\hbox{$E$} \!\mathord{\left/ {\vphantom {E {h^{2} }}}\right.\kern-0pt} \!\lower0.7ex\hbox{${h^{2} }$}}$$ . Here *E* is the elastic modulus, *A* is the cross-section area of the PD bond, and *h* is the characteristic nodal spacing. A choice of α = $${\raise0.7ex\hbox{${h^{2} }$} \!\mathord{\left/ {\vphantom {{h^{2} } E}}\right.\kern-0pt} \!\lower0.7ex\hbox{$E$}}$$ is dimensionally consistent and could effectively minimize the residue forces, which is equivalent to the inverse dominant stiffness in classical Richardson iteration (Saad 2003). The numerical evidence in Sect. 5.2 confirms that this choice yields robust convergence for the nonlinear PD problem studied.

The proposed method differs fundamentally from an adaptive dynamic relaxation. While both approaches employ iterative updates, the present formulation directly targets the static force balance without introducing artificial inertia or damping. As a result, convergence behavior is governed by the residual reduction rather than the kinetic energy decay. A pseudocode of the RBDR method is shown in Fig. 5.

### An adaptive dynamic relaxation method

Dynamic relaxation (ADR) method is commonly employed to obtain quasi-static solutions of the nonlinear peridynamics governing equations without resorting to implicit solvers. Instead of prescribing a constant damping value, the optimal damping at each iteration is estimated from the lowest system frequency ${\omega}_{n}$ using Rayleigh’s quotient,

27 $$ \omega_{n} = \sqrt {\frac{{\left( {U^{n} } \right)^{T} K_{t} U^{n} }}{{\left( {U^{n} } \right)^{T} U^{n} }}} , c_{n} = 2\omega_{n} $$

where, ${K}_{t}$ is the diagonal approximation of tangent stiffness obtained from incremental force variations; ${U}^{n}$ is the displacement vector at time step *n*; ${c}_{n}$ is the updated damping ratio at time step *n*. This adaptive strategy progressively damps higher-frequency components while guiding the solution smoothly toward equilibrium, thereby minimizing oscillations and overshooting. Convergence is achieved when the artificial kinetic energy approaches zero and the residual forces become negligible. The ADR approach is particularly attractive for peridynamic simulations because it retains the simplicity of explicit formulations, avoids the construction of a global stiffness matrix, and remains effective for highly nonlinear problems involving damage evolution.

## Model verification and results

### Stress concentration around a central circular hole in a plate

To verify the hybrid FE-PD model, the classical stress concentration problem of a plate with a central circular hole is employed as a benchmark. The numerical results obtained from four different modeling approaches are compared: a pure Peridynamics (PD) model, a pure finite element (FE) model solved using ABAQUS, the proposed hybrid FE-PD model incorporating the Modified VL-coupling technique, and the analytical solution for the 2D plane stress case derived from the Kirsch solution. For all static analyses, the newly introduced residual-based dynamic relaxation (RBDR) method is employed as the solver.

The pure PD and hybrid FE-PD models are implemented in MATLAB R2023a, developed by extending the PDMATLAB2D code to accommodate the stress concentration problem. The modifications include the introduction of an 8 mm circular hole at the center of the plate. Incorporation of FE subdomains at the top and bottom regions of the domain was made for the hybrid FE-PD models. The plate spans − 0.04 m to 0.04 m in the *x*-direction and − 0.05 m to 0.05 m in the *y*-direction, with a uniform traction stress of 1 MPa applied at the top and bottom edges of the domain.

The material has a Young’s modulus of 72.0 GPa and a mass density of 2440 kg/m^3^, representing a stiff and relatively lightweight brittle solid. A fracture energy of 3.8 J/m^2^ is adopted, governing the energy required for crack initiation and propagation within the peridynamics framework. The nonlocal horizon size is set to *δ* = 0.001 m, defining the interaction radius for long-range peridynamics forces and damage evolution. For numerical stability and accuracy in the dynamic relaxation simulation, a time step of Δ*t* = 8.7 ×  10^−9^s is employed. The complete set of material, geometric, and computational parameters used in this study is summarized in Table 1.

**Table 1:** Material, geometric, and computational parameters

Parameter	Symbol	Value	Unit
Young’s modulus	*E*	72	GPa
Mass density	*ρ*	2440	kg/m^3^
Fracture energy	*G*_*0*_	3.8	J/m^2^
Horizon size	*δ*	0.001	m
Applied traction stress	*σ*_*0*_	1	MPa
Time step	*Δt*	8.7 × 10^−9^	s
Domain length (x-direction)	*L*_*x*_	− 0.04 to 0.04	m
Domain length (y-direction)	*L*_*y*_	− 0.05 to 0.05	m
Hole diameter	*d*	8	mm
Hole radius	*r*_*i*_	4	mm
Plate width	*W*_*p*_	80	mm
Plate height	*H*_*p*_	100	mm
Analysis type	–	2D Plane Stress	–
Solver	–	RBDR	–
Software (PD & Hybrid)	–	MATLAB R2023a	–
Software (FE only)	–	ABAQUS	–

By Saint–Venant’s principle, the presence of the small central hole does not significantly influence the stress distribution at distances greater than three times the hole radius (*r* > 3*r*_*i*_), and uniform far-field tension is recovered at large radius. This classical Kirsch solution serves as the benchmark for verifying the numerical results obtained from the FE, PD and hybrid FE-PD models implemented in the present study.

The stress concentration plots for all models are presented in Fig. 5.

Figure 6 presents a quantitative comparison of the normalized stress distributions (${\sigma}_{xx}/{\sigma}_{0}$ and ${\sigma}_{yy}/{\sigma}_{0}$) along the horizontal path from the hole edge outward, obtained from the four modeling approaches: the analytical Kirsch solution (Den Hartog 1987), pure Peridynamics (PD) model, ABAQUS FE model, and the proposed hybrid FE–PD model.

At the hole boundary (distance = 0), the tangential stress ${\sigma}_{yy}$ reaches a peak value close to 3.0 in the ABAQUS FE model, consistent with the classical stress concentration factor predicted by the Kirsch solution. The hybrid FE–PD and pure PD models show a noticeably lower peak in the immediate vicinity of the hole, reflecting the well-known PD surface effect: material points located within one horizon distance δ from the hole boundary have a truncated interaction neighborhood, which reduces effective bond stiffness and underestimates the local stress peak. For the present configuration (*δ* = 0.001 m, hole radius *r* = 0.004 m, *δ/r* = 0.25), this surface-induced softening is non-negligible. Away from the hole, all models show a smooth decay toward the far-field value of unity, consistent with Saint–Venant’s principle.

The hybrid FE–PD results show excellent agreement with both the analytical solution and the ABAQUS FE results across the entire domain. The pure PD model also captures the general trend well, though with slightly more scattering values compared to the analytical solutions near the hole boundary due to the nonlocal discretization and the influence of the horizon size *δ*. The ${\sigma}_{xx}$ component correctly approaches zero at the hole surface and near-zero values away from it, as expected analytically. Overall, Fig. 6 validates that the proposed hybrid coupling scheme accurately reproduces the classical stress concentration behavior.

Figure 7 provides a visual, full-field comparison of all three in-plane stress components across the plate domain for the three numerical models: FE (ABAQUS), PD (MATLAB), and the hybrid FE–PD (MATLAB) formulation.

For ${\sigma}_{xx}$ (horizontal normal stress), all three models reproduce the characteristic butterfly-shaped compressive stress lobes around the left and right sides of the hole, with tensile regions developing above and below. The FE and hybrid models show sharp, well-resolved contours, while the PD model captures the same pattern with slightly smoother gradients due to its nonlocal nature.

For ${\sigma}_{yy}$ (vertical normal stress), the expected stress amplification at the top and bottom of the hole (SCF ≈ 3) is clearly visible in all three models as a concentrated red region. The stress decays symmetrically toward the far-field applied value of 1 MPa with increasing distance, and all three models show strong qualitative and quantitative agreement with each other.

For ${\sigma}_{xy}$ (shear stress), the four-lobed shear pattern with alternating positive and negative zones at 45° around the hole is consistently reproduced across all three models. The FE and hybrid models show cleaner contour resolution, while the PD model captures the same antisymmetric distribution with characteristic nonlocal smoothing.

A key observation is that the hybrid FE–PD model successfully bridges both the FE subregions at the top and bottom and the PD region around the hole without any visible stress discontinuity at the coupling interface, confirming the effectiveness of the Modified VL-coupling technique.

Figure 8 is particularly significant as it demonstrates the convergence performance of the newly proposed residual-based dynamic relaxation (RBDR) method applied to the hybrid FE–PD model. Three energy quantities are tracked over simulation time (0–300 μs).

For the kinetic energy, it begins at a peak value of approximately 0.014 J and undergoes rapid, monotonic decay, effectively reaching zero by around 50–75 μs. The smooth, non-oscillatory decay confirms that the RBDR damping parameters are well-calibrated and that the method does not suffer from numerical instabilities.

For the strain energy, it rises from zero and stabilizes asymptotically at approximately 0.063 J, plateauing around 150–200 μs. This stabilization indicates that the internal elastic energy of the system has reached its equilibrium distribution, corresponding to the correct static stress state in the plate.

For the total energy, it converges smoothly to the same plateau value of ~ 0.063 J, confirming global energy conservation and equilibrium. The convergence of total energy to a stable value, coinciding with the extinction of kinetic energy, provides a rigorous verification that the RBDR solver has successfully found the static equilibrium solution without spurious oscillations or divergence.

### Comparison of the RBDR and ADR method

The comparison is carried out based on several convergence metrics, namely the relative residual decay versus iteration number shown in Fig. 9a, total energy evolution including kinetic energy (KE) and strain energy (SE), and the kinetic-to-strain energy ratio (KE/SE), which serves as the primary convergence indicator. The performance of both methods is assessed through these metrics to determine their relative computational efficiency and convergence behavior. The results obtained from the RBDR method regarding the relative residual decay versus iteration number are presented in Fig. 9.

Figure 9 presents the convergence characteristics of the RBDR method, where the relative residual decay plot demonstrates a smooth and monotonic reduction from 10° toward the target thresholds of 1% and 0.1% within approximately 300 iterations. The corresponding energy evolution plot confirms that the strain energy remains stable near 10° J while the kinetic energy is effectively suppressed to the order of 10^−20^ J, and the kinetic-to-strain energy (KE/SE) ratio decreases steadily from approximately 10^−18^ to 10^−24^, collectively indicating well-controlled damping and rapid approach to static equilibrium.

In contrast, Fig. 9 shows that the ADR method requires approximately 25,000 iterations to achieve comparable convergence, representing roughly 80 times more iterations than RBDR. Although the ADR energy evolution similarly shows strain energy dominance with suppressed kinetic energy.

The final KE/SE ratio achieved by ADR reaches approximately 10^−21^, which is several orders of magnitude higher than that obtained by RBDR, further confirming the superior convergence quality of the latter. These results demonstrate that while both methods successfully converge to the static equilibrium solution, the RBDR method offers significantly greater efficiency: ∼80 times fewer iterations and ∼23 times wall-clock time. Although both solvers are matrix-free, ADR incurs additional per-iteration overhead for computing the adaptive damping parameter *c*_*n*_ via Rayleigh’s quotient (Eq. 27), whereas RBDR requires only a single force-assembly. Wall-clock times measured on an AMD Ryzen 7 processor (3.30 GHz, 16 GB RAM) show thatRBDR required approximately 0.128 s per iteration while ADR required approximately 3 s per iteration for the Kirsch problem (same convergence threshold). RBDR completed the Kirsch problem, in a ∼23 times wall-clock speedup, under the same convergence threshold. The ∼80 times iteration reduction and ∼23 times wall-clock speed up together quantify the practical efficiency advantage of RBDR. The RBDR method offers significantly greater computational efficiency and convergence precision, making it particularly advantageous for large-scale or computationally intensive structural analyses.

### Numerical investigation of crack branching

A two-dimensional end notch tension experiment is simulated. The specimen is defined over the domain of − 0.05 m ≤ x ≤ 0.05 m and − 0.03 m ≤ y ≤ 0.03 m. The upper and lower subdomains, each having a vertical height of 0.01 m, are discretized using the finite element (FE) method, while the central portion of the domain is modeled with the peridynamics (PD) formulation, as depicted in Fig. 4. A uniform FE mesh size of 0.00033 m is employed in the finite element regions, whereas the PD subdomain is discretized using particles with a spacing of 0.000165 m.

The overlapping zones between the FE and PD subdomains are defined as no-fail regions. These regions play a critical role in transferring forces, displacements, and energy quantities between the FE and PD domains, ensuring stable and consistent coupling. An important limitation of the current formulation is the behavior when a propagating crack approaches the FE–PD interface: the no-fail region suppresses bond breakage within the coupling zone, which would artificially arrest crack growth if the crack reached this boundary. In the present simulations, the PD domain dimensions were chosen to ensure that crack tips remain confined within the PD subdomain throughout the entire simulation window, avoiding this issue. Extending the formulation to handle crack fronts that reach or cross the interface for example, through adaptive domain relocation or a dynamic interface re-partitioning strategy is identified as a priority for future development. A key motivation for adopting the coupled FE–PD framework is the reduction of computational cost associated with full-domain peridynamic simulations. In the present study, the PD formulation is restricted to the fracture-critical central region, while the surrounding elastic domains are efficiently represented using the finite element method. This selective use of nonlocal modeling significantly reduces the overall number of degrees of freedom compared to a full PD discretization, where the entire domain must be resolved with fine particle spacing to capture bond interactions. Although a detailed quantitative comparison in terms of computational time and memory usage between the PD-only and FE–PD models is not included in this work, the reduction in computational burden is evident from the limited extent of the PD region. A systematic benchmarking study will be carried out in future work to provide a rigorous evaluation of speed-up factors and scalability. A pre-notch is introduced within the PD region to initiate crack propagation. pre-notch length of 0.05 m start at coordinate of (-0.05,0) and end at (0,0). The setup is shown on Fig. 4.

A. Two different models are examined: (1) A full peridynamics (PD-only) model subjected to a tensile loading of 2 MPa with a total simulation time of 4.28 × 10^−5^ s; (2) A hybrid FE–PD–FE model subjected to a tensile loading of 2 MPa with a total simulation time of 4.28 × 10^−5^ s.
B. A hybrid FE–PD model without inertial effects, achieved using the RBDR method, subjected to a tensile loading of 8 MPa with a total simulation time of 3.3 × 10^−4^ s.

The peridynamic horizon size is fixed at *δ* = 0.00155 m for all models to maintain consistency. The material is assumed to behave as a brittle solid with a Young’s modulus of 72.0 GPa and a mass density of 2440 kg/m^3^. A fracture energy of 3.8 J/m^2^ is prescribed to control crack initiation and propagation within the peridynamics framework. For numerical stability and accuracy in the dynamic relaxation analysis, a time step of Δ*t* = 8.7 × 10^−9^ s is employed. All parameters summarize in Table 2 below.

**Table 2:** Geometry, material, and computational parameters for dynamic PD-Only, dynamic hybrid FE–PD, and Quasi-static hybrid FE–PD models

Parameter	Symbol	Dynamic PD-only model	Dynamic hybrid FE–PD model	Quasi-Static Hybrid FE–PD model	Unit
Young’s modulus	*E*	72.0	72.0	72.0	GPa
Mass density	*ρ*	2440	2440	2440	kg/m^3^
Fracture energy	*G* _*0*_	3.8	3.8	3.8	J/m^2^
Horizon size	*δ*	0.00155	0.00155	0.00155	m
Applied tensile stress	*σ* _*0*_	2	2	8	MPa
Time step	Δ*t*	8.7 × 10^−9^	8.7 × 10^−9^	8.7 × 10^−9^	s
Total simulation time	*t*	4.28 × 10^−5^	4.28 × 10^−5^	3.3 × 10^−4^	s
Global domain (x-direction)	*L* _*x*_	− 0.05 to 0.05	− 0.05 to 0.05	− 0.05 to 0.05	m
Global domain (y-direction)	*L*_*y*_	− 0.03 to 0.03	− 0.03 to 0.03	− 0.03 to 0.03	m
FE domain height (Top)	*h*_*FE,top*_	–	0.01	0.01	m
FE domain height (Bottom)	*h*_*FE,bottom*_	–	0.01	0.01	m
PD domain height (Center)	*h*_*PD*_	Full Domain	0.04	0.04	m
FE mesh size	*h_FE*	–	0.00033	0.00033	m
PD particle spacing	Δ*x*_*PD*_	0.000165	0.000165	0.000165	m
Coupling overlap region	–	–	No-Fail Region	No-Fail Region	–
Pre-notch length	*a* _*0*_	0.05	0.05	0.05	m
Pre-Notch Start coordinate	–	(− 0.05, 0)	(− 0.05, 0)	(− 0.05, 0)	m
Pre-Notch End coordinate	–	(0, 0)	(0, 0)	(0, 0)	m
Analysis type	–	Dynamic	Dynamic	Quasi-Static	
Coupling configuration	–	PD-Only	FE–PD	FE–PD	
Solver	–	Explicit dynamic	Explicit dynamic	RBDR	
Software (PD & Hybrid)	–	MATLAB R2023a	MATLAB R2023a	MATLAB R2023a	–

#### Fracture propagation time history for dynamic crack branching

Figure 10 presents the mechanical response fields obtained from both the PD-only and hybrid FE–PD models under loading of 2 MPa at *t* = 4.28 × 10^−5^ s. The logarithmic strain energy density field (log_10_*W*) for the PD-only model reveals a well-defined Y-shaped branching pattern, with high energy concentrations sharply localized at the crack tip and along the two diverging branches,

while the surrounding domain retains negligibly low energy values—a distribution physically consistent with brittle dynamic fracture. The hybrid FE–PD model reproduces this branching topology with notable fidelity within the peridynamics subdomain, though the finite element regions exhibit a more diffuse and grid-structured energy distribution owing to the structured FE mesh and the nature of the modified VL-coupling overlap zones. Minor differences in peak energy magnitudes near the FE–PD interface are attributable to the energy transfer mechanism across the coupling regions, yet these discrepancies remain small and do not compromise the overall fracture prediction.

The crack index field (φ) provides the most direct evidence of fracture behavior. In the PD-only model, a sharp and geometrically clean Y-shaped crack is observed, with a long horizontal propagation path bifurcating into two symmetric branches at approximately ± 20–25° from the horizontal axis, and bond damage values concentrated along the crack path. The hybrid model reproduces the crack path and branching angle with high accuracy within the PD subdomain. The finite element regions display a checkerboard-like artifact in the *φ* field, which is a known numerical consequence of the coupling zone formulation rather than a physical damage indicator, and the crack is correctly confined within the peridynamics domain where the fracture mechanics are governed by bond-based constitutive relationships. The close agreement in crack morphology between both models constitutes strong validation of the hybrid framework for dynamic crack branching simulation.

The stress component fields further support this validation. The ${\sigma}_{xx}$ and ${\sigma}_{yy}$ fields in both models exhibit comparable dynamic stress wave patterns, with alternating tensile and compressive regions radiating outward from the crack tip—characteristic of elastic wave propagation accompanying rapid fracture. The PD-only model produces slightly more irregular stress distributions reflective of the discrete particle nature of peridynamics, while the FE regions of the hybrid model yield smoother gradients consistent with classical continuum finite element behavior. Some stress discontinuity is detectable at the FE–PD interface, but it remains sufficiently small to confirm that the modified VL-coupling formulation transmits stresses adequately. The shear stress field ${\sigma}_{xy}$ exhibits a quadrupole-like pattern centered at the branching point in both models, indicative of the mixed-mode stress state near a propagating and bifurcating crack tip, and the qualitative agreement between models is satisfactory.

#### Energy response analysis

The energy evolution plots presented in Fig. 11 provide quantitative validation of the hybrid model’s performance. The total system energy of the hybrid FE–PD model, shown in panel (a), grows monotonically and nearly quadratically from zero to approximately 5.2 J over the simulation duration. The most significant validation result is presented in panel (b), where the total kinetic energy curves of the PD-only and hybrid FE–PD models with modified VL coupling are plotted together. These two curves are virtually indistinguishable throughout the entire simulation, overlapping with negligible deviation as kinetic energy grows from zero to approximately 5.0 J. This near-perfect agreement demonstrates that the hybrid framework conserves momentum and total energy at the global level with minimal numerical error introduced by the coupling formulation, and that the FE subdomains transmit dynamic loads to the PD region without artificial energy dissipation or generation.

The energy response comparisons presented in Fig. 11 provide a quantitative basis for assessing the relative performance of the modified VL-coupling formulation against the standard VL-coupling scheme, with the pure peridynamics solution serving as the reference benchmark. As demonstrated in panels (a) and (b), the total system energy and total kinetic energy trajectories obtained from the modified VL-coupling hybrid model exhibit markedly closer agreement with the PD-only solution over the full simulation window, with deviations remaining within acceptable numerical tolerance throughout. The standard VL-coupling formulation, by contrast, yields progressive divergence from the reference solution beyond *t* ≈ 20 μs, suggesting the accumulation of interface-induced numerical error under loading. The strain energy evolution presented in panel (e) further reinforces this finding. The modified formulation more accurately reproduces the oscillatory character, peak magnitudes, and temporal locations of strain energy fluctuations associated with discrete bond-failure events, whereas the standard VL-coupling scheme exhibits phase shifts and amplitude discrepancies relative to the benchmark. These results demonstrate that the modified VL-coupling formulation delivers superior energy conservation, reduced interface-induced dissipation, and enhanced fidelity in reproducing the dynamic fracture energetics of the reference peridynamics model, thereby validating its adoption as the preferred coupling strategy for hybrid FE–PD simulations involving crack propagation and branching.

The total strain energy in the hybrid model (Modified VL coupling), panel (c), exhibits pronounced oscillations ranging between approximately 0.15 and 0.35 J with an overall decreasing trend beyond *t* ≈ 15 μs. These oscillations are physically meaningful, reflecting stress wave reflections within the domain and discrete bond-breaking events accompanying crack propagation and branching. The peak strain energy near *t* ≈ 10–15 μs corresponds temporally to the onset of crack branching, after which energy is progressively converted into fracture surface energy and kinetic energy of separating material fragments. Panels (d) and (e) isolate the kinetic and strain energy contributions of the PD subdomain within the hybrid model. The PD kinetic energy reaches approximately 3.2 J — accounting for roughly 60–65% of the total kinetic energy — consistent with the PD domain being the most dynamically active region due to ongoing fracture. The PD strain energy mirrors the oscillatory character of the total strain energy, with irregular fluctuations corresponding to successive waves of bond failure. Panels (f) and (g) show that the FE subdomains contribute approximately 35–40% of total kinetic energy, with a smooth and monotonically increasing profile reflecting undamaged elastic wave propagation, and a comparatively small strain energy of up to 0.10 J with mild oscillations corresponding to stress wave passage.

#### Quasi-static case without inertial effects—RBDR method using the hybrid framework

**Fig. 12:**
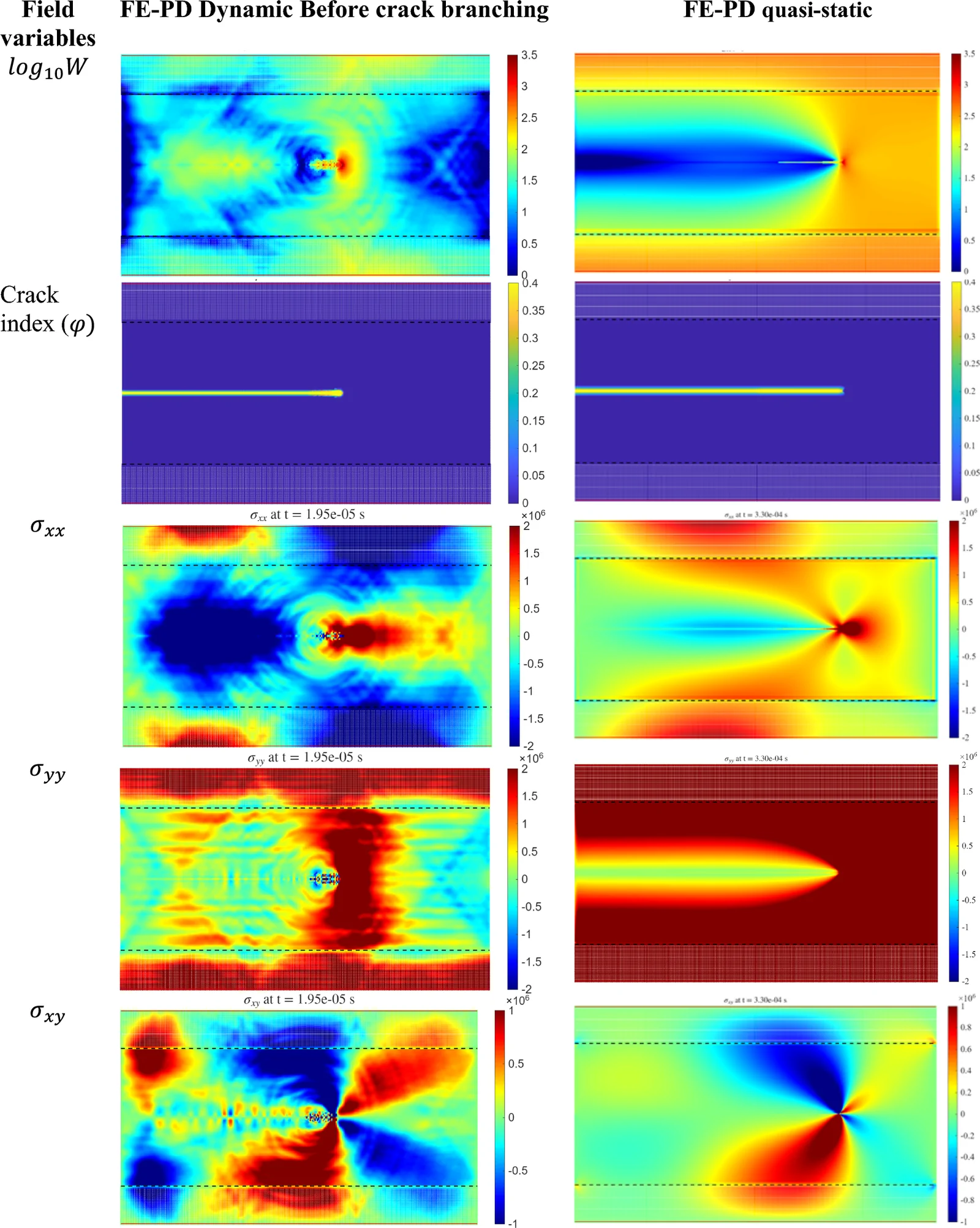
Mechanical response parameters for the hybrid FE–PD models comparing inertial (dynamic, before crack branching, t = 1.95 × 10^−5^ s, 2 MPa) and absence-of-inertial (quasi-static, t = 3.30 × 10^−4^ s, 8 MPa) effects, achieved through the residual-based dynamic relaxation (RBDR) scheme

Figure 12 presents the mechanical response fields comparing the hybrid FE–PD model under dynamic conditions, captured before crack branching at *t* = 1.95 × 10^−5^ s under an applied stress of 2 MPa, against the quasi-static solution obtained via the Residual-based dynamic relaxation (RBDR) scheme at *t* = 3.30 × 10^−4^ s under an applied stress of 8 MPa. As expected from classical fracture mechanics theory, crack branching does not occur under quasi-static conditions, since branching is a well-established inertia-driven instability that requires kinetic energy accumulation at the crack tip a condition that is entirely absent when inertial effects are suppressed (Bobaru and Zhang 2015). The log_10_*W* field in the quasi-static case shows a smooth, elongated elliptical energy lobe concentrated ahead of the crack tip, consistent with the classical Griffith-type strain energy distribution of a slowly propagating Mode-I crack. This energy profile remains qualitatively unchanged regardless of the applied loading magnitude, with only the amplitude scaling accordingly. The crack index (*φ*) confirms a clean, straight, and well-defined crack path, indicative of pure Mode-I fractures under quasi-static conditions. In the dynamic case, by contrast, the log_10_*W* field exhibits a spatially dispersed distribution with oscillatory bands caused by elastic wave propagation and boundary reflections—a direct manifestation of inertial effects. Similarly, the stress field distributions further highlight the fundamental differences between the two loading regimes. In the quasi-static case, *σ*_*xx*_ exhibits a smooth, wedge-shaped tensile stress concentration ahead of the crack tip, consistent with the classical Mode-I singular stress field predicted by Linear Elastic Fracture Mechanics.

*σ*_*yy*_ distribution, however, is dominated by the applied far-field stress of 8 MPa, resulting in a nearly uniform high tensile field throughout the entire specimen with only a minor perturbation near the crack tip a physically expected response under quasi-static loading. The shear stress *σ*_*yy*_ recovers the theoretically anticipated four-quadrant antisymmetric pattern centered precisely on the crack tip, with smooth and well-defined positive and negative shear zones. In the dynamic case, all three stress components are significantly perturbed by elastic wave propagation and boundary reflections. The *σ*_*xx*_ field displays a large compressive zone dominating the domain alongside complex oscillatory patterns around the crack tip, while *σ*_*yy*_ and *σ*_*yy*_ exhibit irregular wave-induced banding throughout the specimen. These perturbations confirm that under dynamic loading, the instantaneous stress state cannot be interpreted independently of the propagating wave field, in stark contrast to the physically consistent and smooth stress distributions recovered under quasi-static conditions.

#### Energy response analysis for the quasi-static case

**Fig. 13:**
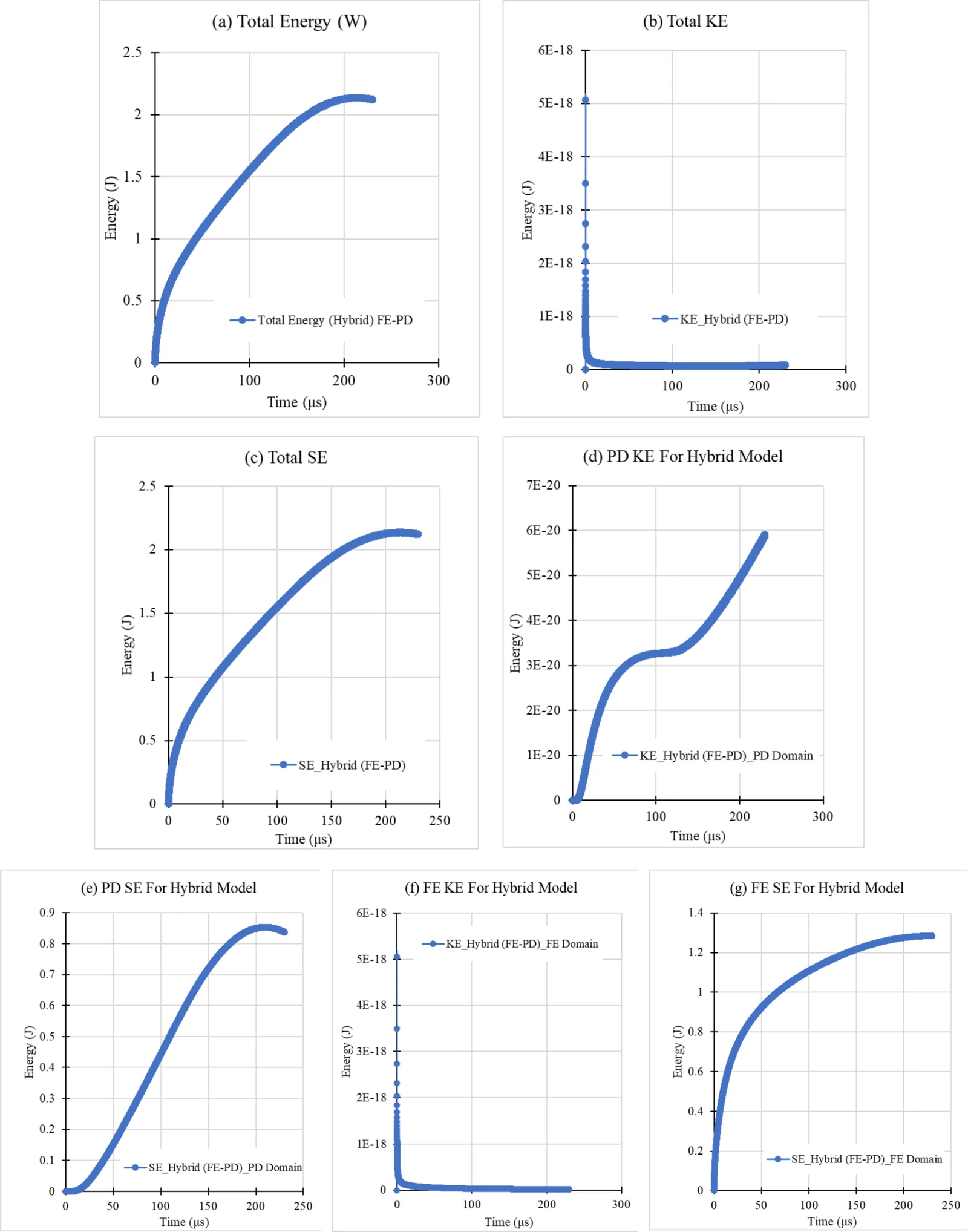
Energy responses of hybrid FE–PD models using the modified VL-coupling formulation under quasi-static conditions: **a** total system energy, **b** total kinetic energy (KE), **c** total strain energy (SE), **d** KE of the PD subdomain, **e** SE of the PD subdomain, **f** KE of the FE subdomains, and **g** SE of the FE subdomains

Figure 13 presents the energy response of the hybrid FE–PD model under quasi-static conditions (8 MPa applied stress). Panel (b) confirms that the RBDR scheme successfully eliminates inertial effects, with the total kinetic energies remaining on the order of 10^−17^ J, effectively zero throughout the simulation, that validates the quasi-static character of the analysis. Since kinetic energy is negligible, the total system energy is entirely composed of strain energy, as confirmed by the close correspondence between panels (a) and (c). The hybrid FE–PD model accumulates a peak total strain energy of approximately 2.15 J at around *t* ≈ 220–250 μs before releasing energy as the crack propagates. The FE coupling zones modify the effective stiffness and energy storage characteristics of the system, with the modified VL-coupling formulation introducing an energy redistribution between subdomains that is more pronounced under quasi-static loading where inertial energy no longer dominates.

The energy partitioning between the PD and FE subdomains of the hybrid model, shown in panels (d) through (g), shows that the FE subdomain strain energy, panel (g), reaches approximately 1.30 J—exceeding the PD subdomain strain energy of approximately 0.85 J in panel (e). Under quasi-static loading, the elastic FE regions therefore store a larger share of total strain energy than the fracturing PD region, a consequence of the local continuum stiffness of the FE subdomains and the energy redistribution introduced by the modified VL-coupling formulation. The smooth, monotonically increasing profiles of both PD and FE strain energies—nonexistence of the oscillations observed in the dynamic case—confirm the absence of wave propagation effects and demonstrate that the RBDR scheme produces a numerically stable and physically consistent energy evolution in the hybrid framework.

#### Comparative assessment of hybrid model performance

The results from the dynamic and quasi-static simulations demonstrate the reliability of the hybrid FE–PD framework across both loading regimes. Under dynamic conditions, the hybrid model reproduces crack branching with near-perfect agreement in global energy evolution, high fidelity in crack morphology, and accurate bifurcation angle and crack path length.

As expected from classical dynamic fracture mechanics theory, the removal of inertial effects entirely suppresses crack branching, reducing the fracture response to a single, straight propagating crack under pure Mode-I conditions. This behavior is confirmed by the hybrid FE–PD model in the quasi-static RBDR simulations even when the applied stress is 8 MPa four times the 2 MPa applied in the dynamic case. The crack in the quasi-static case propagates horizontally from the pre-notch without any angular deflection or bifurcation, and the corresponding strain energy density field shows a smooth, broadly distributed energy concentration ahead of the crack tip, characteristic of classical Griffith fracture. The dynamic model, by contrast, develops the inertia-driven kinetic energy necessary to sustain and amplify crack-tip perturbations beyond the critical branching threshold, leading to the observed bifurcation into two symmetric branches.

The influence of the local–nonlocal coupling is most apparent in the quasi-static regime where inertial contributions no longer dominate the energy budget. In the dynamic case, the overwhelming kinetic energy (~ 5.0 J, over 95% of total system energy) renders any coupling-zone influence on global energy negligible, explaining the near-perfect agreement between PD-only and hybrid energy curves in Sect. 5.3.2. In the quasi-static regime, where total system energy is entirely composed of strain energy (~ 2.15 J), the interaction between the local FE formulation and the nonlocal PD formulation introduces a measurable modification to the effective stiffness. The FE formulation enforces pointwise stress–strain relationships while the PD formulation distributes the material response over the finite horizon δ = 0.00155 m. The modified VL-coupling scheme mediates this through the overlapping no-fail zones, but the energy transfer redistributes strain energy between subdomains: the FE subdomains store approximately 1.30 J compared to 0.85 J in the PD subdomain, with the locally stiffer FE regions accumulating a disproportionately large share of total strain energy under quasi-static loading.

Notwithstanding the energy redistribution between subdomains, the crack path prediction of the hybrid model remains accurate, and the RBDR scheme eliminates inertial effects across both PD and FE subdomains, as confirmed by kinetic energy values on the order of 10^−17^ to 10^−20^ J. The stress field distributions including the *σ*_*xx*_ wedge-shaped tensile stress concentration ahead of the crack tip, the *σ*_*yy*_ near-uniform far-field tensile distribution dominated by the applied loading, and the four-quadrant antisymmetric *σ*_*xy*_ pattern centered on the crack tip are reproduced with excellent fidelity, validating the accuracy of force and displacement transfer through the coupling zones. The quasi-static results highlight both a strength and a current limitation of the hybrid framework: the local–nonlocal coupling accurately transmits kinematic and traction boundary conditions for correct crack path prediction, but the energy partitioning between subdomains is sensitive to the coupling formulation and warrants further refinement. The development of an energy-consistent coupling formulation that enforces energetic equivalence at the local–nonlocal interface is identified as an important direction for future work.

## Conclusions

The following conclusions are drawn from this study:

1. The proposed RBDR solver is a superior alternative to ADR for quasi-static peridynamics analyses. The damping-free, matrix-free Richardson-type solver requires approximately 300 iterations versus approximately 25,000 for ADR (∼80 times fewer iterations), with a wall-clock speedup of approximately 23 times (AMD Ryzen 7, 3.30 GHz), and achieves a final kinetic-to-strain energy ratio of approximately 10^−24^ compared to 10^−21^ for ADR, while requiring no artificial mass or damping tuning. Its compatibility with both standalone PD and hybrid FE–PD coupled systems makes it well-suited for quasi-static fracture simulations.
2. The modified VL-coupling formulation accurately transmits stresses and kinematics across the FE–PD interface, as demonstrated by the Kirsch stress concentration benchmark. The ABAQUS FE model recovers the classical analytical SCF of 3.0 accurately. The hybrid FE–PD and PD-only models reproduce the correct quantitative distribution of the stress concentration, but underestimate the peak stress near the hole boundary due to the PD surface effect arising from the truncated nonlocal interaction horizon near the curved surface; this deviation is a known and physically understood limitation of peridynamics at curved boundaries with non-small δ/r. No spurious stress discontinuities appear at the coupling interface, confirming the effectiveness of the modified VL-coupling scheme. The energy-consistent embedded particle formulation, with four PD particles per interface element, provides a more uniform spatial representation of the coupling zone than single-particle embedding and effectively eliminates artificial force oscillations.
3. The hybrid FE–PD coupling framework accurately replicates full dynamic fracture predictions within the PD subdomain. Under loading, the branching morphology, bifurcation angle, and global energy evolution of the hybrid model are in near-perfect agreement with the PD-only model. The local–nonlocal coupling introduces negligible energy error in the fracture development regime, where the dominant kinetic energy contribution effectively regularizes any coupling-induced discrepancy.
4. The hybrid FE–PD model under quasi-static RBDR loading confirms that crack branching is entirely suppressed in the absence of inertial effects, even at applied stresses four times greater than the dynamic branching threshold. The crack propagates as a single straight Mode-I fracture, and the stress fields are reproduced with excellent fidelity. As expected from classical fracture mechanics theory, crack branching under quasi-static conditions does not occur, and the RBDR solver is validated for quasi-static fracture simulation within the hybrid FE–PD framework.

Extension of the framework to state-based peridynamics would remove the fixed Poisson’s ratio constraint inherent to bond-based formulations and expand applicability to a broader class of engineering materials. Application to three-dimensional fracture problems, including mixed-mode crack growth and fragmentation, represents a natural and important extension of the present two-dimensional hybrid framework.

## Data Availability

All data supporting the findings of this study are available within the paper and its Supplementary Information.
